# Dissecting Breeders’ Sense *via* Explainable Machine Learning Approach: Application to Fruit Peelability and Hardness in Citrus

**DOI:** 10.3389/fpls.2022.832749

**Published:** 2022-02-10

**Authors:** Mai F. Minamikawa, Keisuke Nonaka, Hiroko Hamada, Tokurou Shimizu, Hiroyoshi Iwata

**Affiliations:** ^1^Laboratory of Biometry and Bioinformatics, Department of Agricultural and Environmental Biology, Graduate School of Agricultural and Life Sciences, The University of Tokyo, Tokyo, Japan; ^2^Institute of Fruit Tree and Tea Science, National Agriculture and Food Research Organization (NARO), Shizuoka, Japan

**Keywords:** citrus, breeding, image analysis, machine learning, Bayesian network, deep learning, Grad-CAM

## Abstract

“Genomics-assisted breeding”, which utilizes genomics-based methods, e.g., genome-wide association study (GWAS) and genomic selection (GS), has been attracting attention, especially in the field of fruit breeding. Low-cost genotyping technologies that support genome-assisted breeding have already been established. However, efficient collection of large amounts of high-quality phenotypic data is essential for the success of such breeding. Most of the fruit quality traits have been sensorily and visually evaluated by professional breeders. However, the fruit morphological features that serve as the basis for such sensory and visual judgments are unclear. This makes it difficult to collect efficient phenotypic data on fruit quality traits using image analysis. In this study, we developed a method to automatically measure the morphological features of citrus fruits by the image analysis of cross-sectional images of citrus fruits. We applied explainable machine learning methods and Bayesian networks to determine the relationship between fruit morphological features and two sensorily evaluated fruit quality traits: easiness of peeling (Peeling) and fruit hardness (FruH). In each of all the methods applied in this study, the degradation area of the central core of the fruit was significantly and directly associated with both Peeling and FruH, while the seed area was significantly and directly related to FruH alone. The degradation area of albedo and the area of flavedo were also significantly and directly related to Peeling and FruH, respectively, except in one or two methods. These results suggest that an approach that combines explainable machine learning methods, Bayesian networks, and image analysis can be effective in dissecting the experienced sense of a breeder. In breeding programs, collecting fruit images and efficiently measuring and documenting fruit morphological features that are related to fruit quality traits may increase the size of data for the analysis and improvement of the accuracy of GWAS and GS on the quality traits of the citrus fruits.

## Introduction

The global demand for high-quality fruits is increasing rapidly, and fruit quality has become an essential breeding target (Jenks and Bebeli, 2011). Cross-breeding to obtain cultivars with high-quality fruits generally takes many years due to the long juvenile period of fruit trees. In light of this constraint, it is sensible for the breeders to evaluate as many genotypes as possible to increase the acquisition rate of the new varieties. However, the large size of the fruit trees, makes this to be difficult due to limited orchard space. To overcome these barriers of conventional fruit tree breeding, “genomics-assisted breeding,” which utilizes genomic-based methods, such as genome-wide association study (GWAS) and genomic selection (GS), has been attracting attention, especially in the field of fruit breeding (Iwata et al., 2016). The GWAS can detect and identify quantitative trait loci (QTL) or the genes responsible for the trait of interest without the need for segregating biparental populations required for QTL mapping (Korte and Farlow, 2013). The GS can be used to select superior genotypes in the very early stage of seedlings based on genomic estimated breeding values (GEBV) predicted from genome-wide marker information (Meuwissen et al., 2001). For GWAS and GS to be successful, the phenotypic and marker genotype data should be routinely collected from the real breeding populations. Especially in fruit trees, collecting large datasets obtained from experimental trials is difficult due to the barriers previously listed, and this is in line with the idea of, “breeding-assisted genomics” proposed by Poland (2015). The immense potential of GWAS and GS using real breeding populations has already been reported in fruit trees, e.g., citrus (Minamikawa et al., 2017; Imai et al., 2019), apple (Muranty et al., 2015; Minamikawa et al., 2021), and Japanese pear (Minamikawa et al., 2018; Nishio et al., 2021).

To further improve the accuracy of GWAS and GS for practical breeding, we need to increase both the number and quality of the phenotypic and marker genotype data (Poland, 2015; Iwata et al., 2016; Varshney et al., 2021). While the throughput and cost-effectiveness of genotyping have improved significantly, the measurement of traits of interest, such as fruit quality, remains insufficient in these regards. Most of the fruit quality traits have been subjected to visual and sensory evaluations by a handful of professional breeders, and phenotypic values of the traits are expressed as qualitative categorical scores. For example, in citrus breeding programs, the color of pericarp (nine categories) and the number of seeds (four categories) are traits that are evaluated visually, while the easiness of peeling (Peeling; five categories) and fruit hardness (FruH; five categories) are traits that are subjected to sensory evaluation (Minamikawa et al., 2017). A qualitative assessment based on the sense of the breeder may not be sufficient to evaluate the diverse and continuous variation of the fruit qualities. Expertise in visual and sensory evaluation can be obtained only after a long technical experience, and increasing the number of specialized breeders may not be suitable for low-cost phenotyping.

Image analysis is one way to overcome the shortcomings of the current qualitative evaluation, and it has been applied to quantitative evaluation of fruit quality traits, such as the fruit color of apricot (Farina et al., 2010) and fruit shapes of sweet orange (Costa et al., 2009) and apple (Currie et al., 2000). Although this method can be easily applied to the visually evaluated traits, it is difficult to apply to the sensorily evaluated traits, e.g., Peeling and FruH (Minamikawa et al., 2017), because the fruit morphological features that serve as the basis for such sensory judgments are unclear.

Explainable machine learning methods would be a clue to reveal the relationship between sensorily evaluated traits and the fruit morphological features. In recent years, with improved computer performance and access to big data, various machine learning models have been employed to achieve precision agriculture (e.g., yield prediction and disease detection; Liakos et al., 2018). While traditional machine learning models, such as multiple linear regression (MLR) and random forest (RF), require a feature extraction from images by a specialist, deep neural networks, called deep learning models, perform the feature extraction during the learning process. These machine learning models have been considered as “black boxes” because of the difficulty in interpreting their complex models, but in recent years, various feature interpretation and visualization methods have been proposed. For example, variable importance (Breiman, 2001a), partial dependence for the variable (Friedman, 2001), and variable interactions (Basu et al., 2018) provided an opportunity to interpret the relevance of the features in the generated RF model. Gradient-weighted class activation mapping (Grad-CAM; Selvaraju et al., 2017) visualizes the important features and regions of an image through classification based on deep learning models.

Bayesian networks may also be an effective method to identify the underlying network structure in the sensorily evaluated traits and the fruit morphological features. A Bayesian network is a graphical model that represents the probabilistic relationships among the variables of interest (Heckerman et al., 1995). Yu et al. (2019) used the Bayesian network approach to elucidate the genetic interdependence of various agronomic traits in rice, and predicted the potential influence of external interventions or selections associated with the traits of interest in the interrelated complex traits system.

In this study, we developed a method to quantitatively and automatically evaluate the fruit morphological features using the image analysis of the cross-sectional images of fruits among a wide range of citrus fruits, one of the most cultivated and produced fruits globally (Omura and Shimada, 2016). Then, using the explainable machine learning methods and the Bayesian networks, we investigated the relationship between fruit morphological features and the two fruit quality traits, Peeling and FruH, that were subjected to sensory and qualitative evaluation (Minamikawa et al., 2017), to identify the important fruit morphological features as the sensory indicators of breeders for these fruit quality traits. Peeling and FruH are pivotal traits to affect the freshness and storability of citrus fruits, respectively. Finally, we discussed the similarities and differences between the two highly correlated fruit quality traits (correlation coefficient (*r*) between Peeling and FruH was 0.76; Minamikawa et al., 2017).

## Materials and Methods

### Fruit Harvest and Acquisition of Fruit Images

**FIGURE 1:**
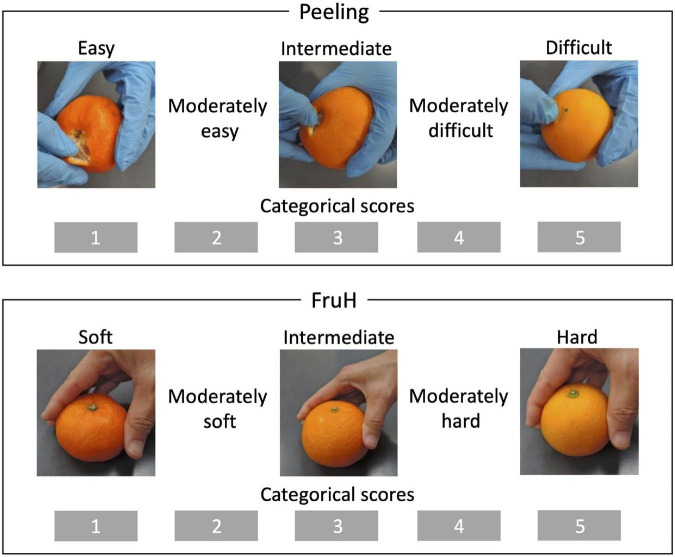
Breeder-evaluated fruit quality traits. Peeling and FruH indicate the easiness of peeling and fruit hardness, respectively. Both the traits were sensorily and qualitatively evaluated by a small number of professional breeders.

**FIGURE 2:**
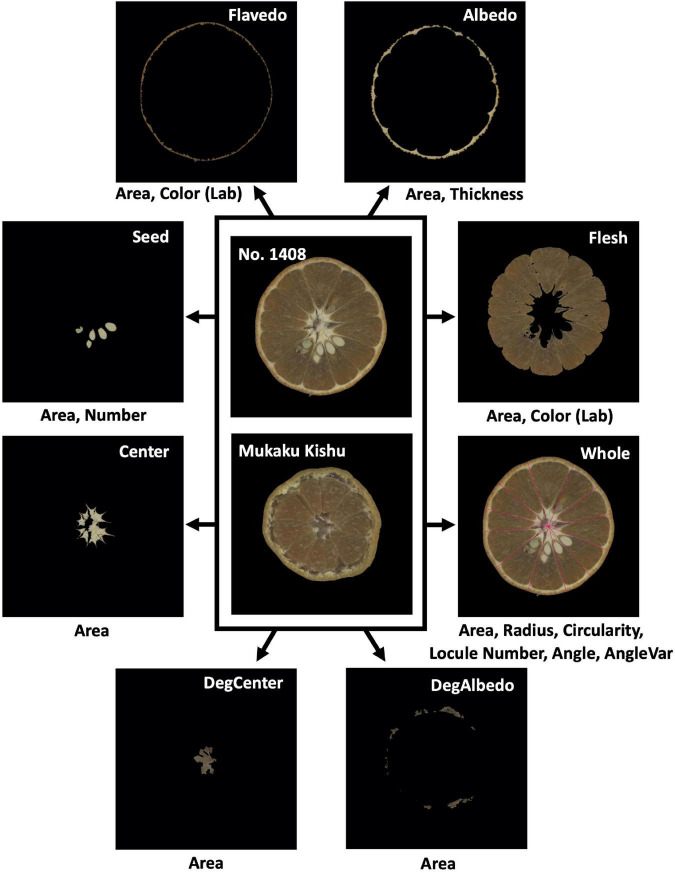
Cross-sectional images of a citrus fruit used for the quantitative evaluation of morphological features. In each image, the top-right information indicates the fruit morphological region (DegCenter = degradation of center; DegAlbedo = degradation of albedo); the bottom information indicates the features evaluated from the morphological region. In total, 21 morphological features (Table 1) were extracted and quantitatively evaluated from the images of citrus fruits using Python.

A total of 108 citrus varieties, a wide range of species, including varieties that are economically cultivated in Japan and used as parents in the breeding program of the National Institute of Fruit and Tea Science (NIFTS; Shizuoka, Japan), were used in this study (Supplementary Table 1). All the varieties were maintained at the NIFTS. The citrus fruits were sampled every December from 2008 to 2014 as described by Minamikawa et al. (2017). Five fruits per variety were obtained from 2008 to 2014 and were used for the evaluation of the two fruit quality traits, the easiness of peeling (Peeling) and the fruit hardness (FruH) (Figure 1; Minamikawa et al., 2017). Furthermore, another five fruits were obtained in 2013 from each of the 92 of these varieties, and again in 2014 from 105 varieties (there were 89 common varieties in both 2013 and 2014); these were used to acquire fruit images (Supplementary Table 1). Due to the nature of the alternative bearing of citrus fruits (2-year cycle of large and small harvests), we could not obtain enough fruits for some varieties in both the years to acquire images. We took 7,020 × 10,200 pixel images containing 10 double-sided (in the case of 2013) or 5 single-sided (in the case of 2014) fruit cross-sections per image from the five fruits of each variety using a flatbed scanner (DS-50000, Epson, Japan). The resolution of the image was 600 dpi with 24-bit colors. The images were then separated into one fruit cross-section, each (Figure 2).

### Evaluation of Fruit Morphological Features Using Image Analysis

Twenty-one morphological features (e.g., areas and colors) of eight regions of each fruit were quantitatively measured by the image analysis (Table 1, Figure 2, and Supplementary Table 2). In instances where some regions of a fruit were not able to be clearly extracted, a characteristic value of the affected regions was set to 0. The method of quantitative evaluation is summarized in Supplementary Table 2. Two image processing libraries in the programming language, Python ver. 3.7.4, “OpenCV” ver. 4.2.0 (Bradski, 2000) and “Scikit-image” ver. 0.15.0 (Van Der Walt et al., 2014), coupled with three data science libraries in Python, “NumPy” ver. 1.17.2 (Harris et al., 2020), “SciPy” ver. 1.4.1 (Virtanen et al., 2020), and “Pandas” ver. 0.25.1 (McKinney, 2010), were used for the image analysis. The mean values for each cultivar were estimated by fitting a mixed linear model (MLM). In the model, the effects of the year and genotype were treated as fixed and random effects, respectively, to remove the yearly effect. The best linear unbiased prediction (BLUP) values of the genotypic effect estimated by the MLM were used as the expected phenotypic values (i.e., genotypic values) of each cultivar in the subsequent analyses. The MLM was implemented in the “lmer” function of the R package “lme4” ver. 1.1–26 (Bates et al., 2015).

**TABLE 1:** Fruit morphological features evaluated in this study.

Region	Feature	Abbreviation
Whole	Whole area of fruit cross-section	Whole area
	Radius of whole area	Radius
	Circularity of whole area	Circularity
	Number of locules in whole area	Locule number
	Central angle of locules in whole area	Locule angle
	Variance of central angle of locules in whole area	Locule angleVar
Flavedo	Flavedo area in whole area^a^	Flavedo area
	Flavedo color (L* value of L*a*b* color space)	Flavedo Lab (L)
	Flavedo color (a* value of L*a*b* color space)	Flavedo Lab (a)
	Flavedo color (b* value of L*a*b* color space)	Flavedo Lab (b)
Albedo	Albedo area in Whole area^a^	Albedo area
	Thickness of Albedo area^b^	Albedo thickness
DegAlbedo	Degradation area of Albedo^a^	DegAlbedo area
Flesh	Flesh area in whole area^a^	Flesh area
	Flesh color (L* value of L*a*b* color space)	Flesh Lab (L)
	Flesh color (a* value of L*a*b* color space)	Flesh Lab (a)
	Flesh color (b* value of L*a*b* color space)	Flesh Lab (b)
Seed	Seed area in Whole area^a^	Seed area
	Seed number in Whole area	Seed number
Center	Central core area in fruit cross-section^a^	Center area
DegCenter	Degradation area of Center area^a^	DegCenter area

*^a^Each value of these areas was divided by the value of the whole area.*

*^b^The value of albedo thickness was divided by the value of the radius. Twenty-one fruit morphological features derived from eight different regions of citrus fruit were quantitatively evaluated using Python (Supplementary Table 2).*

### Breeder-Evaluated Fruit Quality Traits

Easiness of peeling (Peeling) and fruit hardness (FruH) were sensorily and qualitatively evaluated by the breeders (Figure 1; Minamikawa et al., 2017) and scored as five ordinal categories. However, the continuous BLUP values for each cultivar were calculated by fitting the same form of MLM as the fruit morphological features to remove the year effect. The BLUP values were used for subsequent analysis as the expected phenotypic values of each cultivar.

### Multiple Linear Regression and Random Forest

To evaluate the relationships between the two fruit quality traits (Peeling and FruH) and the fruit morphological features, we built models predicting the former based on the latter using two supervised machine-learning algorithms, multiple linear regression (MLR) and non-linear Random Forest (RF) regression. Each fruit quality trait was used as the response variable and the fruit morphological features were used as explainable variables in the machine learning models. To prevent multicollinearity, only 18 of 21 fruit morphological characteristics, which had correlation coefficients between the features of less than 0.95, were used in the constructions of the model. For example, Whole area and Radius (*r* = 0.98), Flavedo Lab (L) and Flavedo Lab (b) (*r* = 0.97), and Albedo area and Albedo thickness (*r* = 0.99) were highly correlated morphological characteristics (Supplementary Figure 1); therefore, one characteristic was randomly selected from each pair of the two characteristics, i.e., Whole area, Flavedo Lab (L), and Albedo area. The MLR can model the response with a linear combination of variables, while RF uses non-linear combinations of variables, and can handle complex interactions among the variables (Breiman, 2001b). For estimating the coefficients in MLR, the R function “lm” (Wilkinson and Rogers, 1973; Chambers, 1992) was used. Although RF has been referred to as a “black box” due to the difficulty in interpreting a model (Breiman, 2001b), we employed the following three indices: variable importance (Breiman, 2001a), partial dependence for the variable (Friedman, 2001), and variable interactions (Basu et al., 2018), which allowed us to interpret the model visually. The variable importance was calculated using the R package, “Boruta” ver. 7.0.0 (Kursa and Rudnicki, 2010), which is specifically designed as a wrapper algorithm built for a RF implementation with the R package, “randomForest” (Breiman, 2001a). The Boruta algorithm can find important variables significantly relevant to the response by comparing the importance of real variables with those of dummy variables, the so-called shadow variables, which were obtained by permuting a copy of the real variables (Kursa and Rudnicki, 2010). The partial dependence plot was generated using the R package, “randomForest” ver. 4.6.14 (Breiman, 2001a). The variable interactions were searched for by using the R package, “iRF” ver. 2.0.0. (Basu et al., 2018). The iRF algorithm can find low- to high-ordered stable interactions of variables by growing weighted random forests iteratively. The stable variable interactions for the select iterations were obtained by analyzing feature usage on the decision paths of large leaf nodes.

To evaluate the goodness of fit of the MLR and RF models, we conducted 10-fold cross-validation (CV) using our 108 citrus varieties. The CV was repeated five times, and the same partition patterns were adapted to the two models in each CV. The prediction accuracy was defined as Pearson’s correlation coefficients (*r*) and root-mean-square error (RMSE) between the predicted and observed values.

### Partial Correlation Analysis

A partial correlation analysis (Kim, 2015) was performed to measure the direct correlation between each of the fruit morphological features and the two fruit quality traits. The fruit morphological features whose associations with the fruit quality traits were significant in either the MLR or the Boruta analysis were used in the partial correlation analysis. The partial correlation coefficient between the two traits was estimated after eliminating the effects of all other fruit morphological features and compared with the correlation coefficient of the apparent correlation which included the influence of the other fruit morphological features. The partial correlation and apparent correlation coefficients were calculated by using the R package, “ppcor” ver. 1.1 (Kim, 2015) and the R standard function, “cor,” respectively.

### Bayesian Network

To estimate the underlying network structure of the fruit morphological features and the breeder-evaluated fruit quality traits (Peeling and FruH), a Bayesian network was constructed. We used the fruit morphological features that were significantly correlated with the fruit quality traits in the partial correlation analysis to construct the Bayesian network. There are three types of algorithms for learning the structure of the Bayesian network: constraint-based algorithm, score-based algorithm, and a hybrid algorithm of these two (Scutari and Denis, 2021). The score-based algorithms have been reported to be more accurate than the constraint-based algorithm because the score-based algorithms, unlike the constraint-based algorithm, consider the whole network structure at once and thus are less sensitive to individual failures (Koller and Friedman, 2009). Furthermore, the hybrid algorithms are more accurate than both the constraint- and score-based algorithms (Tsamardinos et al., 2006). We, therefore, adopted the two score-based algorithms (Hill Climbing and Tabu) and two hybrid algorithms (Max-Min Hill Climbing and General 2-Phase Restricted Maximization) for the construction of Bayesian networks in this study. These algorithms were implemented in the R package, “bnlearn” ver. 4.6.1 (Scutari and Denis, 2021). Prior information on the data, such as that elicited from experts, could be integrated into all the provided algorithms using this package. We assumed that the breeder-evaluated fruit quality traits (Peeling and FruH) were the endpoint of each network and set-up the “blacklist” function in the package to prevent arcs leading away from the fruit quality traits toward the fruit morphological features.

The quality of the network structure was evaluated by the bootstrap resampling and model averaging across 5,000 replications using the “bnlearn” package. The strength of each arc and the confidence of the direction of each arc in the network were estimated probabilistically by using the bootstrapping replicates. The accuracy of the networks was evaluated using the Bayesian information criterion (BIC) score. In the “bnlearn” package, the BIC score is rescaled by –2; that is, a higher BIC value indicates a more accurate structure.

### Deep Learning Models and Feature Visualization

Based on the expected phenotypic values of the fruit quality traits, the fruit images were labeled as shown in Supplementary Table 3 for the binary classification (i.e., easy and difficult classes for Peeling and soft and hard classes for FruH) with deep learning. The number of fruit images to train the deep learning model for each trait was more extensive in 2013 (800 images) than in 2014 (480 images). These fruit images were resized to 224 × 224 pixels to fit with the original image size from the standard image dataset, ImageNet (Deng et al., 2009), and then were augmented by flipping horizontally and vertically and rotating (90°) in the deep learning framework, “Keras” ver. 2.4.3 (Chollet, 2017). In this study, four convolution neural network (CNN) models pretrained with the ImageNet dataset, VGG16, ResNet50, InceptionV3, and InceptionResNetV2, were used for the classification task. All of the models were implemented in the framework, “Keras” (Chollet, 2017). We adopted four fine-tuning strategies (Supplementary Figure 2) for model training. The layers, FT0, FT1, FT2, and FT3 indicate the number of layers (or modules) we chose to fine-tune, while the rest of the layers were frozen (Supplementary Figure 2). The following parameter setting was applied for all the models: the optimizer was Adam, a learning rate of 0.00001, batch size of 30, and the number of epochs was set to 50. The fruit images obtained from 2013 and 2014 were learned separately under the consideration of yearly differences in the fruit quality traits and features in the fruit images. The learned model was saved when the loss value for the validation dataset was the minimum value in 50 epochs, and then evaluated with a confusion matrix and area under the curve (AUC) of a receiver operating characteristics (ROC) plot (Fawcett, 2006) by using the prediction dataset (Supplementary Table 3).

Grad-CAM, a feature visualization method (Selvaraju et al., 2017), was used to visualize the features in the fruit images that were important for the classification of Peeling (easy, difficult) and FruH (soft, hard). Grad-CAM uses the feature map of the last convolutional layer in the model to find the important features in the image. In this study, the last convolutional layer of the simple CNN model, the VGG16, was used for Grad-CAM, as it showed greater classification performance (Akagi et al., 2020). In that study, it was thought to be hard to backpropagate the layers in CNN with more complicated layers, such as ResNet50, InceptionV3, and InceptionResNetV2. The Grad-CAM method was implemented in the deep learning framework, “Keras” ver. 2.4.3 (Chollet, 2017).

The relevance of the classification given by the Grad-CAM method was quantified in each of the seven regions of the fruit (except Whole region) (Table 1 and Figure 2). The mean values of the relevant levels in each region were calculated from the fruit images of the prediction dataset (Supplementary Table 3) that gave correct predictions. Then, the relevance levels were compared between the two classes for both Peeling and FruH to statistically reveal the difference of the features that were important for the classification.

## Results

### Modeling of Fruit Quality Traits Using Fruit Morphological Features

Twenty-one fruit morphological features derived from eight regions of a citrus fruit were quantitatively evaluated for each cultivar with image analysis (Table 1 and Figure 2). To prevent multicollinearity, we chose 18 out of 21 of the fruit morphological features (*r* < 0.95; Supplementary Figure 1) as the explainable variables of the model predicting the fruit quality traits (Peeling and FruH; Figure 1).

**FIGURE 3:**
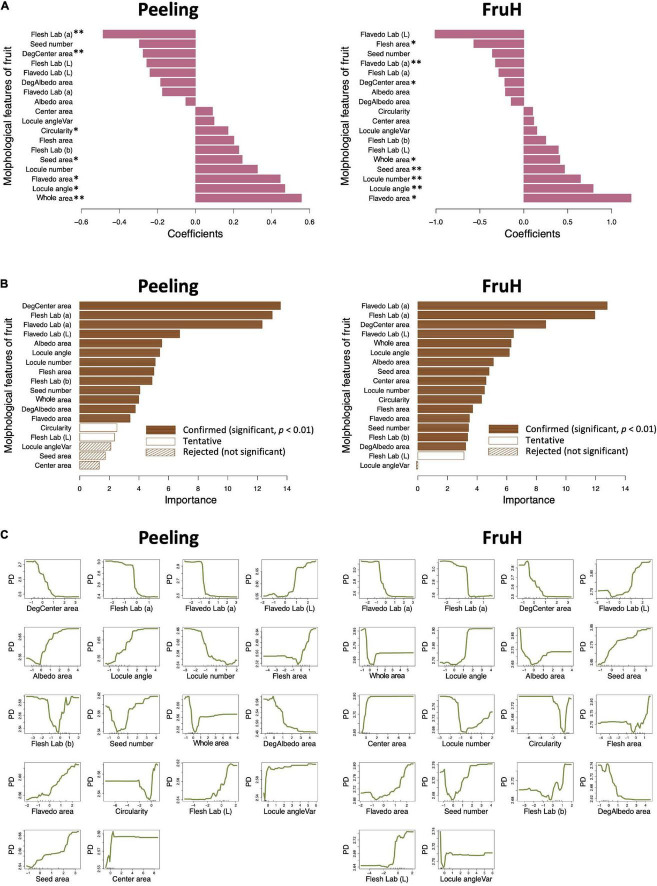
Associations between fruit morphological features and breeder-evaluated fruit quality traits using multiple linear regression and random forest. **(A)** Regression coefficients estimated using multiple linear regression (MLR). Asterisks indicate statistically significant correlations: *p < 0.05; ^**^p < 0.01. **(B)** Variable importance in the random forest (RF) model. **(C)** Partial dependence calculated for the RF model.

Seven [Flesh Lab (a), DegCenter area, Circularity, Seed area, Flavedo area, Locule angle, and Whole area; in the increasing order of regression coefficient] and eight [Flesh area, Flavedo Lab (a), DegCenter area, Whole area, Seed area, Locule number, Locule angle, and Flavedo area; in the increasing order of regression coefficient] fruit morphological features were significantly associated with Peeling and FruH, respectively, in the MLR (Figure 3A and Supplementary Table 4). In contrast, 13 fruit morphological features [DegCenter area, Flesh Lab (a), Flavedo Lab (a), Flavedo Lab (L), Albedo area, Locule angle, Locule number, Flesh area, Flesh Lab (b), Seed number, Whole area, DegAlbedo area, and Flavedo area; in the decreasing order of variable importance] were significantly associated with Peeling, and 16 features [Flavedo Lab (a), Flesh Lab (a), DegCenter area, Flavedo Lab (L), Whole area, Locule angle, Albedo area, Seed area, Center area, Locule number, Circularity, Flesh area, Flavedo area, Seed number, Flesh Lab (b), and DegAlbedo area; in the decreasing order of variable importance] with FruH, in RF (Figure 3B and Supplementary Table 4). DegCenter (degradation of center) area showed a significant association with the two fruit quality traits in both the MLR and RF and had a negative effect on both the traits in MLR (Figure 3A). The partial dependence of the two fruit quality traits was also gradually decreased as the value of the DegCenter area was larger (Figure 3C). In contrast, the Locule angle, Flavedo area, and Whole area were significantly associated with the two fruit quality traits in both the MLR and RF and had positive effects on both the traits in the MLR (Figure 3A). The partial dependence of the two fruit quality traits gradually increased as the values of the Locule angle and Flavedo area were getting larger, while that for Whole area was gradually decreased by as much as around 0 in the value of Whole area, and gradually increased thereafter. Similar trends of association in MLR and RF were observed for the 18 fruit morphological characteristics, including Radius, Flavedo Lab (b), and Albedo thickness, which were not randomly selected in the model constructions to prevent multicollinearity (Supplementary Figure 3). Several low- to high-ordered stable interactions, including DegCenter and Whole area, were detected for both Peeling and FruH by iterative RF (Supplementary Figure 4). Ten-fold CV showed that RF had a higher accuracy (larger *r* and smaller RMSE values) compared to MLR in both the Peeling and FruH (Table 2).

**TABLE 2:** Prediction accuracy of multiple linear regression (MLR) and random forest (RF).

Trait	Correlation			RMSE		
	MLR	RF	*p*-value	MLR	RF	*p*-value
Peeling	0.71 (0.02)	**0.72 (0.01)**	0.30	0.68 (0.02)	**0.66 (0.01)**	0.06
FruH	0.68 (0.02)	**0.70 (0.01)**	0.10	0.77 (0.02)	**0.74 (0.01)**	**0.04**

*The prediction accuracy was measured as Pearson’s correlation coefficient (r) and root mean square error (RMSE) for the predicted and observed values. Values in bold signify the greatest accuracy (the highest r and the lowest RMSE values) and p-values < 0.05 for each trait. Values in parentheses represent the SD.*

**FIGURE 4:**
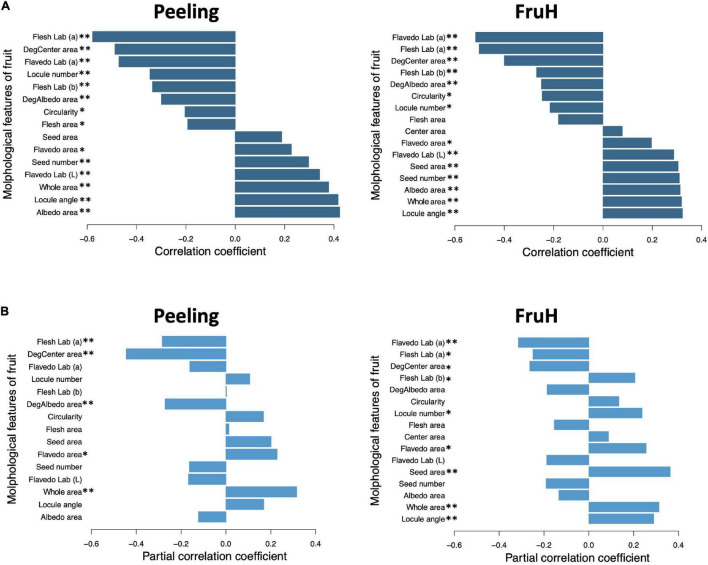
Apparent and partial correlations between fruit morphological features and breeder-evaluated fruit quality traits. **(A)** Apparent correlation coefficients. **(B)** Partial correlation coefficients. Asterisks indicate statistically significant correlations: *p < 0.05; ^**^p < 0.01.

Flesh Lab (a) and Flavedo Lab (a) were significantly associated with Peeling and FruH, respectively, in both the MLR and RF (Figures 3A,B). Both the features had larger negative effects on the two fruit quality traits, although it is unlikely that this color information is directly associated with the physical properties of Peeling and FruH. We, therefore, performed a partial correlation analysis to measure the direct correlation between each of the fruit morphological features and the two fruit quality traits. The partial correlation coefficients between Flesh Lab (a) and Peeling and between Flavedo Lab (a) and FruH largely decreased, compared with the apparent correlation (Figure 4). Excluding the characteristics related to color, DegCenter area was significantly correlated with Peeling and FruH in the partial correlation and had the largest negative effect on the two fruit quality traits (Figure 4B and Supplementary Table 4). In contrast, Whole area was significantly correlated with the two fruit quality traits and had the largest and the second-largest positive effects on Peeling and FruH, respectively. Seed area, which was significantly correlated with only FruH, had the largest positive effect on FruH.

Satsuma mandarin (*Citrus unshiu* Marcov.), which showed the lowest Peeling and FruH values (i.e., easiest Peeling and softest FruH) among the varieties used in this study, had higher or lower values for DegCenter and Whole areas, respectively, among the varieties (Supplementary Figure 5). On the other hand, Banpeiyu (*Citrus maxima* Merr.) showed the opposite trend to that of Satsuma. For Seed area, Satsuma (softest FruH) and Banpeiyu (the third hardest FruH) had lower and higher values, respectively, among the varieties.

### Construction of Network Structures for Fruit Morphological Features and Fruit Quality Traits *via* Bayesian Networks

**FIGURE 5:**
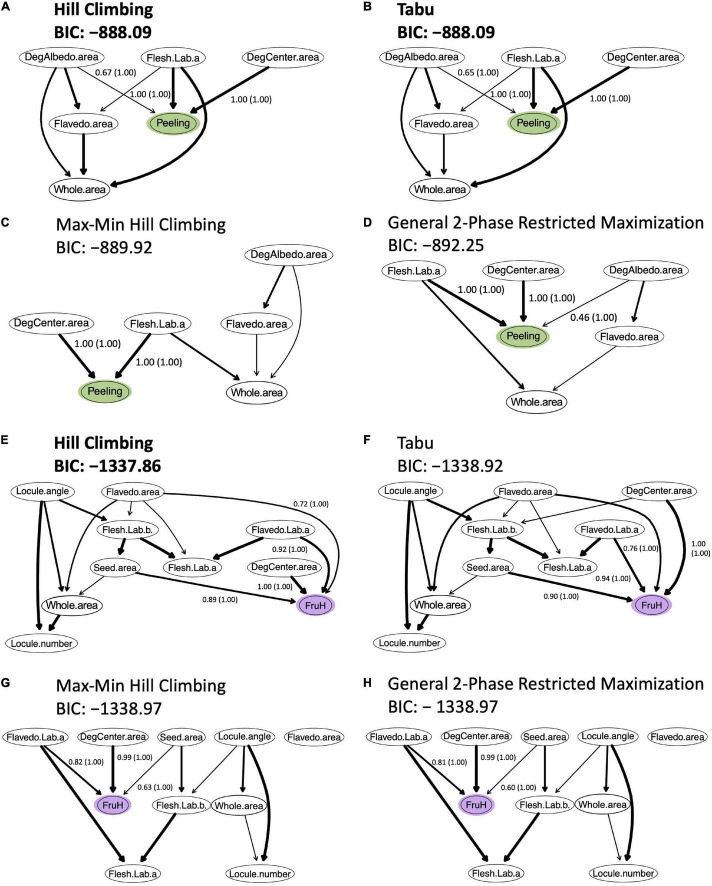
Bayesian networks for causal inference between fruit morphological features and breeder-evaluated fruit quality traits. **(A–D)** Bayesian networks for the Peeling. **(E–H)** Bayesian networks for the FruH. For each trait, four algorithms were trialed. Two of these were score-based [Hill Climbing **(A,E)** and Tabu **(B,F)**]; the other two were hybrid algorithms [Max-Min Hill Climbing **(C,G)** and General 2-phase restricted maximization **(D,H)**]. Arc thickness represents arc strength. Labels provide the strength (and in parentheses, confidence regarding direction) of arcs; these are only shown for those arcs connecting features with traits. For each trait, the algorithms with the highest Bayesian information criterion (BIC) score are highlighted in bold. A higher BIC score is better because the BIC score is rescaled by –2 in the “bnlearn” package.

To build the Bayesian network, the five (DegCenter area, Flesh Lab (a), DegAlbedo area, Flavedo area, and Whole area; in the increasing order of partial correlation coefficient) and nine (Flavedo Lab (a), DegCenter area, Flesh Lab (a), Flesh Lab (b), Locule number, Flavedo area, Locule angle, Whole area, and Seed area; in the increasing order of partial correlation coefficient) fruit morphological features, which were all significantly associated with Peeling and FruH, respectively, in the partial correlation analysis, were used. The two score-based algorithms (Hill Climbing and Tabu) produced a greater number of arcs and showed higher accuracies (higher BIC values) than the two hybrid algorithms (Max-Min Hill Climbing and General 2-Phase Restricted Maximization) (Figures 5A–H). Both Hill Climbing and Tabu, which returned the largest BIC scores, showed the most favorable networks for Peeling (Figures 5A,B). Hill Climbing was also the best network for FruH (Figure 5E).

Among the most favorable networks for Peeling, DegCenter and DegAlbedo areas and Flesh Lab (a) had directed arcs toward Peeling (Figures 5A,B and Supplementary Table 4). The arc strength was greater for DegCenter area and Flesh Lab (a) (1.00) than DegAlbedo area (<0.7). The confidence for the arc direction was also high for all the three morphological features. On the other hand, DegCenter, Seed, Flavedo areas, and Flavedo Lab (a) had directed arcs toward FruH in the most favorable network (Figure 5E and Supplementary Table 4). Arc strength was the greatest for DegCenter area (1.00). The confidence for the arc direction was also high for all the four morphological features. Although the Whole area was significantly associated with Peeling and FruH, and had a larger positive effect on the two fruit quality traits, it did not have a directed edge to Peeling and FruH in any of the favored Bayesian networks (Figures 5A,B,E). The whole area was shown to be indirectly associated with Peeling and FruH.

### Visualization of Fruit Morphological Features That Contribute to Classification Using Deep Learning Models

Among the four CNN models applied in this study, InceptionV3 attained the highest classification accuracy for Peeling (0.94) and FruH (0.95), respectively (Table 3), although even the simplest CNN, VGG16 (Supplementary Figure 2), showed almost the same accuracy as InceptionV3 for each trait (0.92 for Peeling and 0.95 for FruH) (Table 3 and Supplementary Figures 6–9). The ROC-AUC values also supported this trend (Supplementary Figures 6C, 7C, 8C, 9C). Classification accuracy was higher when using the 2013 images than those from 2014 (Table 3). The FT1, FT2, and FT3 strategies offered higher accuracies compared to FT0 for both the two fruit quality traits and years. The accuracy either improved or worsened as more FT layers (or modules) were added for ResNet50 and VGG16, respectively; however, the difference in the performance among FT1, FT2, and FT3 strategies was not so clear for other models. We observed some misclassifications for fruit images with phenotypic values from 1.5 to 3.4 in the prediction dataset (Supplementary Figure 10).

**TABLE 3:** Classification accuracy of deep learning models.

Trait	Year	VGG16				ResNet50				InceptionV3				InceptionResNetV2			
		FT0	FT1	FT2	FT3	FT0	FT1	FT2	FT3	FT0	FT1	FT2	FT3	FT0	FT1	FT2	FT3
Peeling	2013	0.85	**0.92**	0.88	0.92	0.69	0.79	0.81	**0.86**	0.91	**0.94**	**0.94**	**0.94**	0.90	**0.93**	0.88	0.89
	2014	0.76	**0.90**	0.88	0.86	0.66	0.78	**0.84**	0.76	0.86	**0.90**	0.78	0.78	0.88	0.86	0.86	**0.90**
FruH	2013	0.85	**0.95**	**0.95**	**0.95**	0.65	0.73	0.78	**0.81**	0.88	0.92	0.90	**0.95**	0.79	0.88	**0.89**	0.87
	2014	0.84	**0.90**	0.86	0.88	0.58	0.70	0.68	**0.74**	0.78	0.80	0.76	**0.86**	0.80	**0.82**	**0.82**	0.88

*Different fine-tuning strategies (FT0, FT1, FT2, and FT3; see Supplementary Figure 2) were compared. Values in bold signify the highest accuracy among the models for each trait and year.*

**FIGURE 6:**
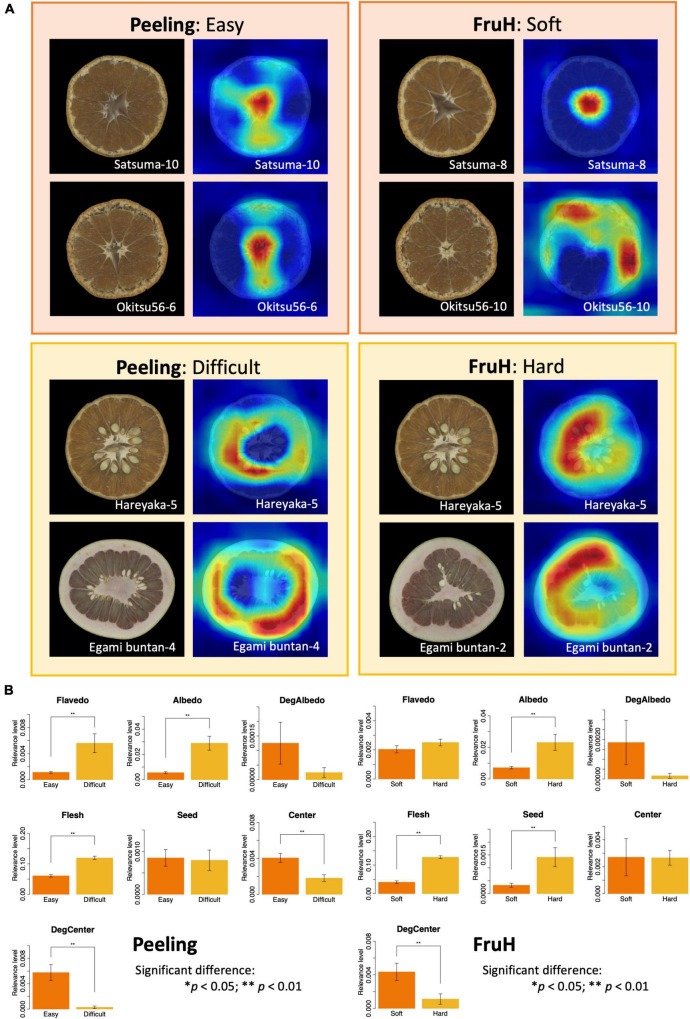
Visualization of fruit morphological features contributing to deep learning-based classification. **(A)** Grad-CAM visualization using the last convolutional layer of the VGG16 model. For each trait, the left and right images indicate the original and feature-visualized images, respectively. **(B)** Differences in the relevance levels between the classification classes for each fruit morphological region. Asterisks indicate statistically significant differences: *p < 0.05; **p < 0.01.

Grad-CAM visualization revealed the key features in the fruit image that contribute to the classification. The central and albedo degradation areas were found to be more relevant to the easy and soft classes for Peeling and FruH, respectively, than the difficult and hard classes for the two fruit quality traits (Figure 6A). In contrast, the flesh and albedo areas showed higher relevance in both the difficult and hard classes for Peeling and FruH, respectively. The seed area also appeared to be highly relevant, especially with respect to the hard class for FruH.

The relevance levels of the seven regions of a fruit (except Whole region) (Table 1 and Figure 2) were quantified using the prediction dataset (Supplementary Table 3) to statistically reveal the difference between the two classes of each fruit quality trait (Figure 6B and Supplementary Table 4). The area of the DegCenter region had significantly higher relevance levels in the easy and soft classes for Peeling and FruH, respectively. On the other hand, the areas of Albedo and Flesh regions showed significantly higher relevance levels in the difficult and hard classes for the two fruit quality traits. The areas of Flavedo and Seed regions showed significantly high levels of relevance only in the difficult and hard classes of Peeling and FruH.

## Discussion

In this study, we developed a method to quantitatively and automatically evaluate fruit morphological features by using the image analysis of cross-sectional fruit images of citrus fruits. We then derived 21 fruit morphological features from 8 regions of the fruit. Multiple explainable machine learning methods and Bayesian networks highlighted the fruit morphological features that were important as sensory indices for the breeder-evaluated traits, Peeling and FruH.

Random forest attained a higher prediction accuracy than the MLR for both the fruit quality traits. The result may be explained by the fact that RF can incorporate the non-linear relationships between a response variable (i.e., a fruit quality trait) and an explainable variable (i.e., fruit morphological features), as well as the influences of complex interactions among the explainable variables (Basu et al., 2018). We found that there were complex patterns in the partial dependencies (e.g., Whole area) indicating the non-linear relationships between fruit morphological features and the fruit quality traits, as well as the influences of several low- to high-ordered stable interactions among the fruit morphological features. The superiority of RF over MLR has also been reported for yield predictions of wheat, maize, and potato (Jeong et al., 2016). The high performance of RF in these instances was said to be due to capturing the influences of interactions among environmental variables, including climate, soil, photoperiod, water, and fertilization data (Jeong et al., 2016).

Bayesian networks estimated the underlying network structure in the fruit morphological features and the breeder-evaluated fruit quality traits. The result that two score-based algorithms were more accurate than two hybrid algorithms is consistent with the recent findings from the studies of wheat (Momen et al., 2021) and rice (Yu et al., 2019). It may be useful to genetically improve the fruit morphological features that have directed (rather than undirected) arcs to Peeling and/or FruH to improve these fruit quality traits. However, a caution is required when interpreting the causal relationships estimated by the networks, because Bayesian networks have many assumptions (Heckerman et al., 1995), including that these networks were constructed by only the observed variables and did not include any unobserved variables. Flesh Lab (a) and Flavedo Lab (a) were directly connected to Peeling and FruH, respectively, implying the existence of unobserved variables between these variables, as information on color is not likely to be directly associated with the physical properties of Peeling and FruH. The partial correlation coefficients of these color information variables, after removing the influence of other observed variables, are not zero, which may also support this hypothesis. To elucidate the detailed relationship between these color information variables and the fruit quality traits, it would be better to increase the number of citrus varieties and explainable variables by extracting and evaluating more fruit morphological features from other image types (e.g., longitudinal section).

The higher classification accuracy of deep learning models when using the images of 2013 may be explained by the fact that we had more fruit images from 2013 than from 2014. In general, a large dataset has been required for the success of deep learning (Lecun et al., 2015). FT1, FT2, and FT3 strategies offered higher accuracies compared to FT0, confirming the importance of fine-tuning strategy (Chollet, 2017). However, the VGG16 with more layers for the fine-tuning, led to worse results. The fine-tuned VGG16 may overfit to the dataset and learn irrelevant features in the image due to their large entropic capacity (Chougrad et al., 2018). Some misclassifications were found in the images showing moderate Peeling and FruH values, which might have only small actual differences in the features between the two classes in the moderate levels. Even among the breeders, sensory evaluations can differ, especially among the moderate levels. Thus, there is a possibility that the binary label classification was ambiguous and not accurately assigned in these levels.

By combining the Grad-CAM visualization and the information of the fruit morphological features, the relevance of fruit morphological features to the classification was revealed in an objective manner. This suggests that it would be important to connect the visualization results with knowledge on plant physiology and breeding. Toda and Okura (2019) have stated that “even if the visualization methods generate meaningful results, humans still play the most important role in evaluating the visualization results by connecting the computer-generated results with professional knowledge, for example, in plant science”. The feature visualization methods have provided novel insights into agronomically important traits (e.g., calyx-end cracking (called, *hetasuki* in Japanese; Akagi et al., 2020) and seedlessness (Masuda et al., 2021) of persimmon fruits) at an accelerating pace. The biological interpretation of the visualization results by the physiologists and breeders would be important not only to increase the reliability of deep learning models but also to understand the molecular mechanism of targeted traits.

Integrating all the methods applied in this study, DegCenter area was significantly and directly associated with both Peeling and FruH in all of the methods used, while Seed area was also significantly and directly associated with FruH in each of the methods (Supplementary Table 4). The significant associations between the two morphological features and the two quality traits were practically observed in Satsuma mandarin and Banpeiyu (Supplementary Figure 5). This result suggests that DegCenter area would contribute to the high correlation between the two fruit quality traits, while the difference between the two traits may be explained by Seed area. Citrus fruits with a larger DegCenter area tended to be easier-peeling and softer fruits in this study. On the other hand, citrus fruits with a larger Seed area tended to be harder fruits. To improve these fruit quality traits, it would be effective to change the DegCenter and Seed areas to their desired directions. DegAlbedo and Flavedo areas would be other candidates to change in improving the Peeling and FruH, respectively, because DegAlbedo and Flavedo areas were significantly and directly associated with Peeling and FruH in all but one or two methods (Supplementary Table 4). The citrus fruits with a larger DegAlbedo area and a smaller Flavedo area tended to be easier-peeling and softer fruits. Whole area, which was significantly, but not directly, associated with both Peeling and FruH in all of the methods, could also be a candidate for the improvement of the two fruit quality traits.

Some representative easy-peeling mandarins have been reported to have loose albedo with great aerial spaces (Yu et al., 2021). In citrus fruits with large albedo degradation, called peel puffing, the degradation of the central core also tended to be large (Inoue, 1980). It seems that the degradations of albedo have a connection to the central core. Thus, citrus fruits with a larger DegCenter area could have degradation of albedo (including small amounts that were difficult to be evaluated by image analysis in this study) which may lead to easy peeling. The ease of peeling is also affected by the hardness of citrus fruits, because the breeders must first puncture the peel with their fingers and then separate the peel from the flesh locules (Figure 1). The cavity of the citrus fruit with a large DegCenter area would soften the fruit, and hence would lead to easy peeling. Small Flavedo area would also contribute to a soft fruit and easy peeling. Yu et al. (2021) suggested that total peel thickness, which showed a high correlation with flavedo thickness (*r* = 0.64), was relevant to peel firmness and hence contributed to the ease of peeling, although flavedo, albedo, and total peel thickness were not significantly correlated with the ease of peeling (Goldenberg et al., 2014, 2018).

Metabolome analysis revealed a lower concentration of citric acid in the albedo tissue of the peel puffing citrus fruits (i.e., peelable and soft fruits) than that in the normal citrus fruits (Ibáñez et al., 2014). The GWAS using the parental citrus varieties and breeding population detected a significant common single nucleotide polymorphism (SNP) on Chromosome 4 for acid and weight (Minamikawa et al., 2017). This SNP resided in the gene *Ciclev10031681 m.g*, which is annotated as glutamate dehydrogenase (GDH). The GDH is involved in avocado fruit maturation (Loulakakis et al., 1994). Thus, GDH could have pleiotropic effects on the concentration of citric acid and fruit weight/size, which are involved in citrus fruit maturation. Citric acid is the major organic acid in citrus fruits, and the acidity and weight/size of fruits generally decrease and increase, respectively, during fruit maturation (Omura and Shimada, 2016). Considering the relation of the citric acid with Peeling and FruH (Ibáñez et al., 2014) and GDH that may have pleiotropic effects on citric acid and whole area (i.e., fruit size), the significant association of the whole area with Peeling and FruH observed in this study could be related to the concentration of citric acid, which may be regulated by the GDH.

Cell-wall polysaccharides have been reported to be associated with cell wall development and strength, which influence fruit firmness and peelability of the citrus fruits (Muramatsu et al., 1999; Kita et al., 2000; Minamikawa et al., 2017; Goldenberg et al., 2018). The concentration of cell wall polysaccharides was significantly higher in the flavedo tissue of the hard-peel of Hassaku (*C. hassaku hort.* ex Tanaka) than that in the soft-peel of Satsuma mandarin (*C. unshiu* Marcov.), implying that the degradation of the cell wall polysaccharides resulted in the peel softening (Muramatsu et al., 1999). The expression of cell wall-related genes, endoxyloglucan transferase, expansin, extensin, glycine-rich protein, and pectinacetylesterase homologues, was detected in both the albedo and flavedo tissues, during the citrus fruit development (Kita et al., 2000). However, the expression pattern of three of those genes was different between the albedo and flavedo tissues, which may lead to the formation of large intercellular spaces in the albedo, and hence accelerate the ease of peeling (Kita et al., 2000).

The characteristics of fruit hardness in the presence or absence of seeds have been evaluated in citrus (Sharif et al., 2021), avocado (Hershkovitz et al., 2010, 2011), and atemoya (dos Santos et al., 2019). Fruit firmness was significantly higher in seeded “Kinnow” mandarins than less seeded “Kinnow” strains (Sharif et al., 2021). The seeded avocado fruit has been reported to be more firm compared to the seedless fruit because seeds in the avocado fruit were involved in the delay of the ripening processes (Hershkovitz et al., 2010, 2011). Ethylene application elicited lower levels of ethylene in the seeded fruit than in the seedless fruit, concomitantly with a massive augmentation of a gene coding for a negative regulator of ethylene responses, PaCTR1 (Hershkovitz et al., 2010). This implied that the negative regulator, PaCTR1 may moderate the effect of ethylene on the seeded fruit (Hershkovitz et al., 2010). It has been suggested that ethylene could be implicated also in the regulation of fruit maturation of non-climacteric citrus (Alonso et al., 1995; Katz et al., 2004). In atemoya, the seedless fruits showed less firmness and had a lower content of calcium in the exocarp compared to the seeded fruits (dos Santos et al., 2019). Calcium has been considered to inhibit fruit softening by preserving the cell wall of the fruits (dos Santos et al., 2019).

In this study, we combined the image analysis, explainable machine learning methods, and Bayesian networks to investigate and identify the fruit morphological features that could act as sensory indices for the breeder-evaluated fruit quality traits, Peeling and FruH. The results suggest that the approaches applied in this study could be effective to dissect the “breeder’s sense,” which has been considered up to now as a “black box”. The fruit morphological features relevant to Peeling and FruH could be used as novel indices for Peeling and FruH in a citrus breeding program. The efficient collection of data on fruit morphological features related to fruit quality traits from a breeding program could improve the accuracy of GWAS and GS for citrus fruit quality traits. It can also accelerate both “breeding-assisted genomics” (Poland, 2015) and “genomics-assisted breeding” (Iwata et al., 2016; Varshney et al., 2021), simultaneously, to contribute to citrus breeding and genetics.

## Data Availability Statement

The raw data supporting the conclusions of this article will be made available by the authors, without undue reservation.

## Author Contributions

MFM, KN, HH, TS, and HI conceived and designed this study. KN and HH evaluated the fruit quality traits of citrus and prepared the images of citrus fruit. MFM performed all the statistical analyses used in this study. KN, HH, TS, and HI provided valuable suggestions for the statistical analyses. MFM drafted the manuscript. All authors have read and approved the manuscript.

## Conflict of Interest

The authors declare that the research was conducted in the absence of any commercial or financial relationships that could be construed as a potential conflict of interest.

## Publisher’s Note

All claims expressed in this article are solely those of the authors and do not necessarily represent those of their affiliated organizations, or those of the publisher, the editors and the reviewers. Any product that may be evaluated in this article, or claim that may be made by its manufacturer, is not guaranteed or endorsed by the publisher.
