# Supplementary material for: Dissecting Breeders’ Sense via Explainable Machine Learning Approach: Application to Fruit Peelability and Hardness in Citrus
Source: Front Plant Sci. 2022 Feb 10;13:832749. doi: 10.3389/fpls.2022.832749 (PMC8867066; doi:10.3389/fpls.2022.832749)
Supplement: Supplementary file 1 [file Data_Sheet_1.pdf]

Supplementary Table 1. Citrus varieties used in this study.

No.	Variety name	Strain name	Type	Parentage (seed × pollen) or species name	Reference	Acquisition of fruit images[b]	
						2013	2014
1	Akemi	(stock strain)	Hybrid cultivar	Kiyomi × Seminole	Shimizu et al. (2016)	✓	✓
2	Aki tangor	(stock strain)	Hybrid cultivar	Satsuma × Sweet orange	Shimizu et al. (2016)	✓	✓
3	Amaka	(stock strain)	Hybrid cultivar	Kiyomi × Encore	Shimizu et al. (2016)	✓	✓
4	Amakusa	(stock strain)	Hybrid cultivar	KyOw14 × Page	Nonaka et al. (2017)	✓	✓
5	Ariake	(stock strain)	Hybrid cultivar	Sweet orange × Clementine	Shimizu et al. (2016)	✓	✓
6	Asumi	(stock strain)	Hybrid cultivar	Okitsu 46 × Harumi	Shimizu et al. (2016)	✓	✓
7	Benibae	(stock strain)	Hybrid cultivar	HF9 × Encore	Shimizu et al. (2016)	✓	✓
8	Ehime Kashi No.28	(stock strain)	Hybrid cultivar	Nanko × Amakusa	Shimizu et al. (2016)	✓	✓
9	Encore	(stock strain)	Hybrid cultivar	King × Willowleaf mandarin	Shimizu et al. (2016)	–	✓
10	Fairchild	(stock strain)	Hybrid cultivar	Clementine × Orland	Shimizu et al. (2016)	–	✓
11	Fortune	(stock strain)	Hybrid cultivar	Clementine × Dancy	Shimizu et al. (2016)	✓	✓
12	Harehime	(stock strain)	Hybrid cultivar	E-647 × Satsuma	Shimizu et al. (2016)	✓	✓
13	Hareyaka	(stock strain)	Hybrid cultivar	Encore × Ponkan	Shimizu et al. (2016)	✓	✓
14	Haruhi	(stock strain)	Hybrid cultivar	Okitsu 46 × Awa Orange	Shimizu et al. (2016)	–	✓
15	Harumi	(stock strain)	Hybrid cultivar	Kiyomi × Ponkan	Shimizu et al. (2016)	✓	✓
16	Hayaka	(stock strain)	Hybrid cultivar	Satsuma × Ponkan	Shimizu et al. (2016)	✓	✓
17	Kankitsu Chukanbohon Nou 5 Gou	(stock strain)	Hybrid cultivar	Lee × Kishu	Shimizu et al. (2016)	✓	✓
18	Kankitsu Chukanbohon Nou 6 Gou	(stock strain)	Hybrid cultivar	King × Kishu	Shimizu et al. (2016)	✓	✓
19	Kanpei	(stock strain)	Hybrid cultivar	Nishinokaori × Ponkan	Shimizu et al. (2016)	✓	✓
20	Kara	(stock strain)	Hybrid cultivar	Satsuma × King	Shimizu et al. (2016)	✓	✓
21	Kiyomi	(stock strain)	Hybrid cultivar	Satsuma × Sweet orange	Shimizu et al. (2016)	✓	✓
22	Lee	(stock strain)	Hybrid cultivar	Clementine × Orland	Shimizu et al. (2016)	✓	✓
23	Mihaya	(stock strain)	Hybrid cultivar	Tsunonozomi × No.1408	Shimizu et al. (2016)	✓	✓
24	Mihocore	(stock strain)	Hybrid cultivar	Satsuma × Encore	Shimizu et al. (2016)	✓	✓
25	Nanko	(stock strain)	Hybrid cultivar	Satsuma × Clementine	Shimizu et al. (2016)	✓	✓
26	Nishinokaori	(stock strain)	Hybrid cultivar	Kiyomi × Sweet orange	Shimizu et al. (2016)	✓	✓
27	Nova	(stock strain)	Hybrid cultivar	Clementine × Orland	Shimizu et al. (2016)	–	✓
28	Osceola	(stock strain)	Hybrid cultivar	Clementine × Orland	Shimizu et al. (2016)	✓	✓
29	Page	(stock strain)	Hybrid cultivar	Mineola × Clementine	Shimizu et al. (2016)	–	✓
30	Reiko	(stock strain)	Hybrid cultivar	KyEn5 × Murcott	Nonaka et al. (2017)	✓	✓
31	Robinson	(stock strain)	Hybrid cultivar	Clementine × Orland	Shimizu et al. (2016)	✓	✓
32	Saga mandarin	(stock strain)	Hybrid cultivar	Satsuma × Fairchild	Shimizu et al. (2016)	✓	✓
33	Seiho	(stock strain)	Hybrid cultivar	Kiyomi × Mineola	Shimizu et al. (2016)	✓	✓
34	Seinannohikari	(stock strain)	Hybrid cultivar	KyOw21 × Youkou	Shimizu et al. (2016)	✓	✓
35	Seminole	(stock strain)	Hybrid cultivar	Grapefruit × Dancy	Shimizu et al. (2016)	–	✓
36	Setoka	(stock strain)	Hybrid cultivar	Tsunonozomi × Murcott	Nonaka et al. (2017)	✓	✓
37	Setomi	(stock strain)	Hybrid cultivar	Kiyomi × Ponkan	Shimizu et al. (2016)	✓	✓
38	Shiranuhi	(stock strain)	Hybrid cultivar	Kiyomi × Ponkan	Shimizu et al. (2016)	✓	✓
39	Southern Red	(stock strain)	Hybrid cultivar	Kara × Ponkan	Nonaka et al. (2017)	✓	✓
40	Southern Yellow	(stock strain)	Hybrid cultivar	Tanikawa Buntan × Kishu	Shimizu et al. (2016)	✓	–
41	Sweet Spring	(stock strain)	Hybrid cultivar	Satsuma × Hassaku	Shimizu et al. (2016)	✓	✓
42	Tamami	(stock strain)	Hybrid cultivar	Kiyomi × Willking	Shimizu et al. (2016)	✓	✓
43	Tsunokagayaki	(stock strain)	Hybrid cultivar	KyOw14 × Encore	Shimizu et al. (2016)	✓	✓
44	Tsunokaori	(stock strain)	Hybrid cultivar	Kiyomi × Satsuma	Shimizu et al. (2016)	✓	✓
45	Tsunonozomi	(stock strain)	Hybrid cultivar	Kiyomi × Encore	Shimizu et al. (2016)	✓	✓
46	Wilking	(stock strain)	Hybrid cultivar	King × Willowleaf mandarin	Shimizu et al. (2016)	✓	✓
47	Youkou	(stock strain)	Hybrid cultivar	Kiyomi × Ponkan	Shimizu et al. (2016)	–	✓
48	E-647	(stock strain)	Breeding/selected strains	Kiyomi × Osceola	Shimizu et al. (2016)	✓	✓
49	EnOw21	(stock strain)	Breeding/selected strains	Encore × Satsuma	Shimizu et al. (2016)	–	✓
50	HF9	(stock strain)	Breeding/selected strains	Satsuma × Sweet orange	Shimizu et al. (2016)	✓	✓

Supplementary Table S1. (*Continued*)

No.	Variety	Strain	Type	Parentage or scientific name	Reference	Acquisition of fruit images[b]	
						2013	2014
51	JHG	(stock strain)	Breeding/selected strains	Satsuma × Hyuganatsu	Selected strain C013 in Shimizu et al. (2016)	–	✓
52	Kuchinotsu 18	(stock strain)	Breeding/selected strains	KyOw21 × Encore	Selected strain C019 in Shimizu et al. (2016)	✓	✓
53	Kuchinotsu 27	(stock strain)	Breeding/selected strains	EnOw21 × Youkou	Selected strain C022 in Shimizu et al. (2016)	–	✓
54	Kuchinotsu 28	(stock strain)	Breeding/selected strains	KyOw21 × Dancy	Selected strain C023 in Shimizu et al. (2016)	✓	✓
55	Kuchinotsu 33	(stock strain)	Breeding/selected strains	KyOw14 × Encore	Selected strain C027 in Shimizu et al. (2016)	✓	✓
56	Kuchinotsu 35	(stock strain)	Breeding/selected strains	KyOw21 × Encore	Selected strain C028 in Shimizu et al. (2016)	✓	✓
57	Kuchinotsu 36	(stock strain)	Breeding/selected strains	? × Murcott	Selected strain C029 in Shimizu et al. (2016)	✓	✓
58	Kuchinotsu 38	(stock strain)	Breeding/selected strains	KyOw21 × Robinson	Selected strain C030 in Shimizu et al. (2016)	✓	✓
59	Kuchinotsu 39	(stock strain)	Breeding/selected strains	Encore × Satsuma	Selected strain C031 in Shimizu et al. (2016)	✓	✓
60	Kuchinotsu 40	(stock strain)	Breeding/selected strains	KyOw21 × ?	Selected strain C032 in Shimizu et al. (2016)	–	✓
61	Kuchinotsu 49	(stock strain)	Breeding/selected strains	Kuchinotsu 38 × ?	Selected strain C037 in Shimizu et al. (2016)	✓	✓
62	Kuchinotsu 51	(stock strain)	Breeding/selected strains	? × Kuchinotsu 27	Selected strain C038 in Shimizu et al. (2016)	✓	✓
63	Kuchinotsu 52	(stock strain)	Breeding/selected strains	Tsunokagayaki × Ariake	Selected strain C039 in Shimizu et al. (2016)	✓	✓
64	KyEn4	(stock strain)	Breeding/selected strains	Kiyomi × Encore	Selected strain C040 in Shimizu et al. (2016)	✓	✓
65	KyEn5	(stock strain)	Breeding/selected strains	Kiyomi × Encore	Selected strain C041 in Shimizu et al. (2016)	✓	✓
66	KyOw14	(stock strain)	Breeding/selected strains	Kiyomi × Satsuma	Shimizu et al. (2016)	✓	✓
67	KyOw21	(stock strain)	Breeding/selected strains	Kiyomi × Satsuma	Shimizu et al. (2016)	✓	✓
68	KyOw21xAriake22	(stock strain)	Breeding/selected strains	KyOw21 × Ariake	Selected strain C042 in Shimizu et al. (2016)	✓	✓
69	KyOw21xD4	(stock strain)	Breeding/selected strains	KyOw21 × Dancy	Selected strain C043 in Shimizu et al. (2016)	–	✓
70	LeeAo9	(stock strain)	Breeding/selected strains	Lee × Satsuma	Selected strain C044 in Shimizu et al. (2016)	✓	✓
71	M5	(stock strain)	Breeding/selected strains	Kiyomi × Robinson	Selected strain C045 in Shimizu et al. (2016)	✓	✓
72	No.1011	(stock strain)	Breeding/selected strains	Nankou × 2700xOIy25	Selected strain C047 in Shimizu et al. (2016)	✓	✓
73	No.1408	(stock strain)	Breeding/selected strains	EnOw21 × No.2681	Shimizu et al. (2016)	✓	✓
74	Okitsu 46	(stock strain)	Breeding/selected strains	Sweet Spring × Sweet orange	Shimizu et al. (2016)	✓	✓
75	Okitsu 56	(stock strain)	Breeding/selected strains	Okitsu 45 × Kankitsu Chukanbohon Nou 5 Gou	Shimizu et al. (2016)	✓	–
76	Okitsu 57	(stock strain)	Breeding/selected strains	Okitsu 46 × Harumi	Selected strain C069 in Shimizu et al. (2016)	✓	✓
77	Okitsu 59	(stock strain)	Breeding/selected strains	A7 × F118	Selected strain C070 in Shimizu et al. (2016)	✓	✓
78	Okitsu 60	(stock strain)	Breeding/selected strains	Okitsu 46 × Harumi	Selected strain C071 in Shimizu et al. (2016)	✓	✓
79	Okitsu 62	(stock strain)	Breeding/selected strains	Harehime × Okitsu 56	Selected strain C073 in Shimizu et al. (2016)	✓	✓
80	Okitsu 63	(stock strain)	Breeding/selected strains	E-647 × Ponkan	Selected strain C074 in Shimizu et al. (2016)	✓	✓
81	Okitsu 67	(stock strain)	Breeding/selected strains	Harehime × Okitsu 56	Selected strain C077 in Shimizu et al. (2016)	✓	✓
82	SBxHg	(stock strain)	Breeding/selected strains	Satsuma × ?	Selected strain C082 in Shimizu et al. (2016)	✓	✓
83	u-22	(stock strain)	Breeding/selected strains	Harehime × Asumi	Selected strain C084 in Shimizu et al. (2016)	✓	✓
84	980389	(stock strain)	Breeding/selected strains	Unknown	Selected strain C005 in Shimizu et al. (2016)	✓	✓
85	031045	(stock strain)	Breeding/selected strains	Harehime × Clementine	Selected strain C002 in Shimizu et al. (2016)	–	✓
86	Banpeiyu	(stock strain)	Indigenous variety	*C. maxima* Merr.	Shimizu et al. (2016)	✓	✓
87	Clementine	(stock strain)	Indigenous variety	*C. clementina* hort. ex Tanaka	Shimizu et al. (2016)	–	✓
88	Dancy	(stock strain)	Indigenous variety	*C. tangerina* hort. ex Tanaka	Shimizu et al. (2016)	✓	✓
89	Egami buntan	(stock strain)	Indigenous variety	*C. maxima* Merr.	Shimizu et al. (2016)	✓	✓
90	Grapefruit	Triumph[a]	Indigenous variety	*C. paradisi* Macfad.	Shimizu et al. (2016)	✓	✓
91	Haruka	(stock strain)	Indigenous variety	Hyuganatsu × Natsudaidai	Shimizu et al. (2016)	✓	✓
92	Hassaku	(stock strain)	Indigenous variety	*C. hassaku* hort. ex Tanaka	Shimizu et al. (2016)	✓	✓
93	Hirado buntan	(stock strain)	Indigenous variety	*C. maxima* Merr.	Shimizu et al. (2016)	✓	✓
94	Hyuganatsu	(stock strain)	Indigenous variety	*C. tamurana* hort. ex Tanaka	Shimizu et al. (2016)	✓	✓
95	Iyo	Miyauchi	Indigenous variety	*C. iyo* hort. ex Tanaka	Shimizu et al. (2016)	✓	✓
96	Kawachi bankan	(stock strain)	Indigenous variety	*C. maxima* Merr.	Shimizu et al. (2016)	✓	✓
97	King	(stock strain)	Indigenous variety	*C. nobilis* Lour.	Shimizu et al. (2016)	–	✓
98	Kishu	Mukaku Kishu (seedless Kishu)	Indigenous variety	*C. kinokuni* hort. ex Tanaka	Shimizu et al. (2016)	✓	✓
99	Mato buntan	(stock strain)	Indigenous variety	*C. maxima* Merr.	Shimizu et al. (2016)	✓	–
100	Mediterranean mandarin	(stock strain)	Indigenous variety	*C. deliciosa* Ten.	Shimizu et al. (2016)	–	✓
101	Murcott	(stock strain)	Indigenous variety	*C.* spp	Shimizu et al. (2016)	✓	✓
102	Natsudaidai	Kawano	Indigenous variety	*C. natsudaidai* Hayata	Shimizu et al. (2016)	✓	✓
103	Ponkan	Yoshida[a]	Indigenous variety	*C. reticulata* Blanco	Shimizu et al. (2016)	✓	✓
104	Satsuma	Okitsu[a]	Indigenous variety	*C. unshiu* Marcov.	Shimizu et al. (2016)	✓	✓
105	Soren tangelo	(stock strain)	Indigenous variety	*C.* spp	Shimizu et al. (2016)	✓	✓
106	Sweet orange	Trovita[a]	Indigenous variety	*C. sinensis* (L.) Osbeck	Shimizu et al. (2016)	✓	✓
107	Tankan	Tarumizu1gou[a]	Indigenous variety	*C. tankan* Hayata	Shimizu et al. (2016)	✓	✓
108	Yuge hyoukan	(stock strain)	Indigenous variety	*C. yuge-hyokan* hort. ex Yu.Tanaka	Shimizu et al. (2016)	✓	✓

[a]Triumph, Yoshida, Okitsu, Trovita, and Tarumizu1gou were used as representative mutants of Grapefruit, Ponkan, Satsuma, Sweet orange, and Tankan, respectively.

[b]Five fruits were obtained in 2013 from each of the 92 of 108 varieties, and again in 2014 from 105 varieties (there were 89 common varieties in both 2013 and 2014); these were used to acquire fruit images.

References

・Nonaka, K., Fujii, H., Kita, M., Shimada, T., Endo, T., Yoshioka, T., et al. (2017). Identification and parentage analysis of citrus cultivars developed in Japan by CAPS markers. Hortic. J. 86, 208–221. doi:10.2503/hortj.OKD-026.

・Shimizu, T., Kitajima, A., Nonaka, K., Yoshioka, T., Ohta, S., Goto, S., et al. (2016). Hybrid origins of citrus varieties inferred from DNA marker analysis of nuclear and organelle genomes. PLoS One 11(11), e0166969. doi:10.1371/journal.pone.0166969.

Supplementary Table 2. Method of quantitative evaluation for morphological features of citrus fruit.

Region	Feature	Abbreviation	Summary of quantitative evaluation
Whole	Whole area of fruit cross-section	Whole area	The area in an image with L^* value ($L^*a^*b^*$ color space) > 0.
	Radius of Whole area	Radius	The mean of the radius value at h (horizontal axis) = 0 in Whole regions of the images obtained by the affine transformation from 1 to 360 degrees in increments of 1 degree.
	Circularity of Whole area	Circularity	Circularity was defined as $4\pi \times$ (Whole area) / (perimeter of Whole region)^2.
	Number of locules in Whole area	Locule number	The locules were divided by the relative (local) maxima of Albedo thickness.
	Central angle of locules in Whole area	Locule angle	The mean of the central angles for each locule in Whole region.
	Variance of central angle of locules in Whole area	Locule angleVar	The variance of the central angles for each locule in Whole region.
Flavedo	Flavedo area in Whole area[a]	Flavedo area	The outer pericarp area in Whole region with $L^* \leq 110$ for $129.45 \leq a^*$, $L^* \leq 120$ for $127.95 \leq a^* < 129.45$, or $L^* \leq 130$ for $a^* < 127.95$.
	Flavedo color (L^* value of $L^*a^*b^*$ color space)	Flavedo Lab (L)	The mean of L^* in each pixel of Flavedo region. L^* indicates whiteness (higher value) or blackness (smaller value).
	Flavedo color (a^* value of $L^*a^*b^*$ color space)	Flavedo Lab (a)	The mean of a^* in each pixel of Flavedo region. a^* indicates redness (higher value) or greenness (smaller value).
	Flavedo color (b^* value of $L^*a^*b^*$ color space)	Flavedo Lab (b)	The mean of b^* in each pixel of Flavedo region. b^* indicates yellowness (higher value) or blueness (smaller value).
Albedo	Albedo area in Whole area[a]	Albedo area	The middle pericarp area in Whole region with $L^* \geq 130$ for $129.45 \leq a^*$, $L^* \geq 140$ for $127.95 \leq a^* < 129.45$, or $L^* \geq 150$ for $a^* < 127.95$.
	Thickness of Albedo area[b]	Albedo thickness	The mean of the thickness values at h (horizontal axis) = 0 in the images of Albedo region obtained by the affine transformation from 1 to 360 degrees in increments of 1 degree.
DegAlbedo	Degradation area of Albedo[a]	DegAlbedo area	The degradation area of the middle pericarp in Whole region with $L^* \leq 110$ for $129.45 \leq a^*$, $L^* \leq 120$ for $127.95 \leq a^* < 129.45$, or $L^* \leq 130$ for $a^* < 127.95$.
Flesh	Flesh area in Whole area[a]	Flesh area	The flesh area in Whole region with $L^* \leq 110$ for $129.45 \leq a^*$, $L^* \leq 120$ for $127.95 \leq a^* < 129.45$, or $L^* \leq 130$ for $a^* < 127.95$.
	Flesh color (L^* value of $L^*a^*b^*$ color space)	Flesh Lab (L)	The mean of L^* values in each pixel of Flesh region.
	Flesh color (a^* value of $L^*a^*b^*$ color space)	Flesh Lab (a)	The mean of a^* values in each pixel of Flesh region.
	Flesh color (b^* value of $L^*a^*b^*$ color space)	Flesh Lab (b)	The mean of b^* values in each pixel of Flesh region.
Seed	Seed area in Whole area[a]	Seed area	The seed area in Whole region with $L^* \geq 130$ for $129.45 \leq a^*$, $L^* \geq 140$ for $127.95 \leq a^* < 129.45$, or $L^* \geq 150$ for $a^* < 127.95$, and circularity ≥ 0.25.
	Seed number in Whole area	Seed number	The number of objects in Seed region.
Center	Central core area in fruit cross-section[a]	Center area	The central core area in Whole region with $L^* \geq 130$ for $129.45 \leq a^*$, $L^* \geq 140$ for $127.95 \leq a^* < 129.45$, or $L^* \geq 150$ for $a^* < 127.95$.
DegCenter	Degradation area of Center area[a]	DegCenter area	The degradation area of the central core area in Whole region with $L^* \leq 110$.

[a] Each value of these areas was divided by the value of Whole area.

[b] The value of Albedo thickness was divided by the value of Radius.

Supplementary Table 3. Summary of the number of fruit images used for deep learning models. Training and Validation datasets were used for training the deep neural networks, and the Prediction dataset was used for the evaluation of the learned model.

Breeder-evaluated traits	Year	Class	The number of images		
			Training	Validation	Prediction
Peeling	2013	Easy	300	100	50
		Difficult	300	100	50
	2014	Easy	180	60	25
		Difficult	180	60	25
FruH	2013	Soft	300	100	50
		Hard	300	100	50
	2014	Soft	180	60	25
		Hard	180	60	25

Supplementary Table 4. Summary of the results obtained from all analyses used in this study.
MLR: multiple linear regression (Figure 3A), RF: random forest (Figure 3B), Apparent correlation (Figure 4A), Partial correlation (Figure 4B), Bayesian network (Figure 5), Grad-CAM (Figure 6). The information on fruit morphological regions was used for Grad-CAM, while the information on fruit morphological features was used for all other analyses. Asterisks indicate statistically significant correlations: *$p < 0.05$; **$p < 0.01$. “ns” indicates $p \geq 0.05$. DC and non-DC indicate direct- and non-direct connections, respectively, from the fruit morphological features to the breeder-evaluated fruit quality traits (Peeling or FruH) in the most reliable network, which returned the largest Bayesian information criterion (BIC) score (Figure 5).

Fruit morphology		Peeling						FruH					
Region	Feature	MLR	RF	Apparent correlation	Partial correlation	Bayesian network	Grad-CAM	MLR	RF	Apparent correlation	Partial correlation	Bayesian network	Grad-CAM
	Whole area	**	**	**	**	non-DC		*	**	**	**	non-DC	
	Circularity	*	ns	*	ns	–		ns	**	*	ns	–	
Whole	Locule number	ns	**	**	ns	–	–	**	**	*	*	non-DC	–
	Locule angle	*	**	**	ns	–		**	**	**	**	non-DC	
	Locule angleVar	ns	ns	–	–	–		ns	ns	–	–	–	
	Flavedo area	*	**	*	*	non-DC		*	**	*	*	DC	
Flavedo	Flavedo Lab (L)	ns	**	**	ns	–	**	ns	**	**	ns	–	ns
	Flavedo Lab (a)	ns	**	**	ns	–		**	**	**	**	DC	
Albedo	Albedo area	ns	**	**	ns	–	**	ns	**	**	ns	–	**
DegAlbedo	DegAlbedo area	ns	**	**	**	DC	ns	ns	**	**	ns	–	ns
	Flesh area	ns	**	*	ns	–		*	**	ns	ns	–	
Flesh	Flesh Lab (L)	ns	ns	–	–	–	**	ns	ns	–	–	–	**
	Flesh Lab (a)	**	**	**	**	DC		ns	**	**	*	non-DC	
	Flesh Lab (b)	ns	**	**	ns	–		ns	**	**	*	non-DC	
Seed	Seed area	*	ns	ns	ns	–	ns	**	**	**	**	DC	**
	Seed number	ns	**	**	ns	–		ns	**	**	ns	–	
Center	Center area	ns	ns	–	–	–	**	ns	**	ns	ns	–	ns
DegCenter	DegCenter area	**	**	**	**	DC	**	*	**	**	*	DC	**

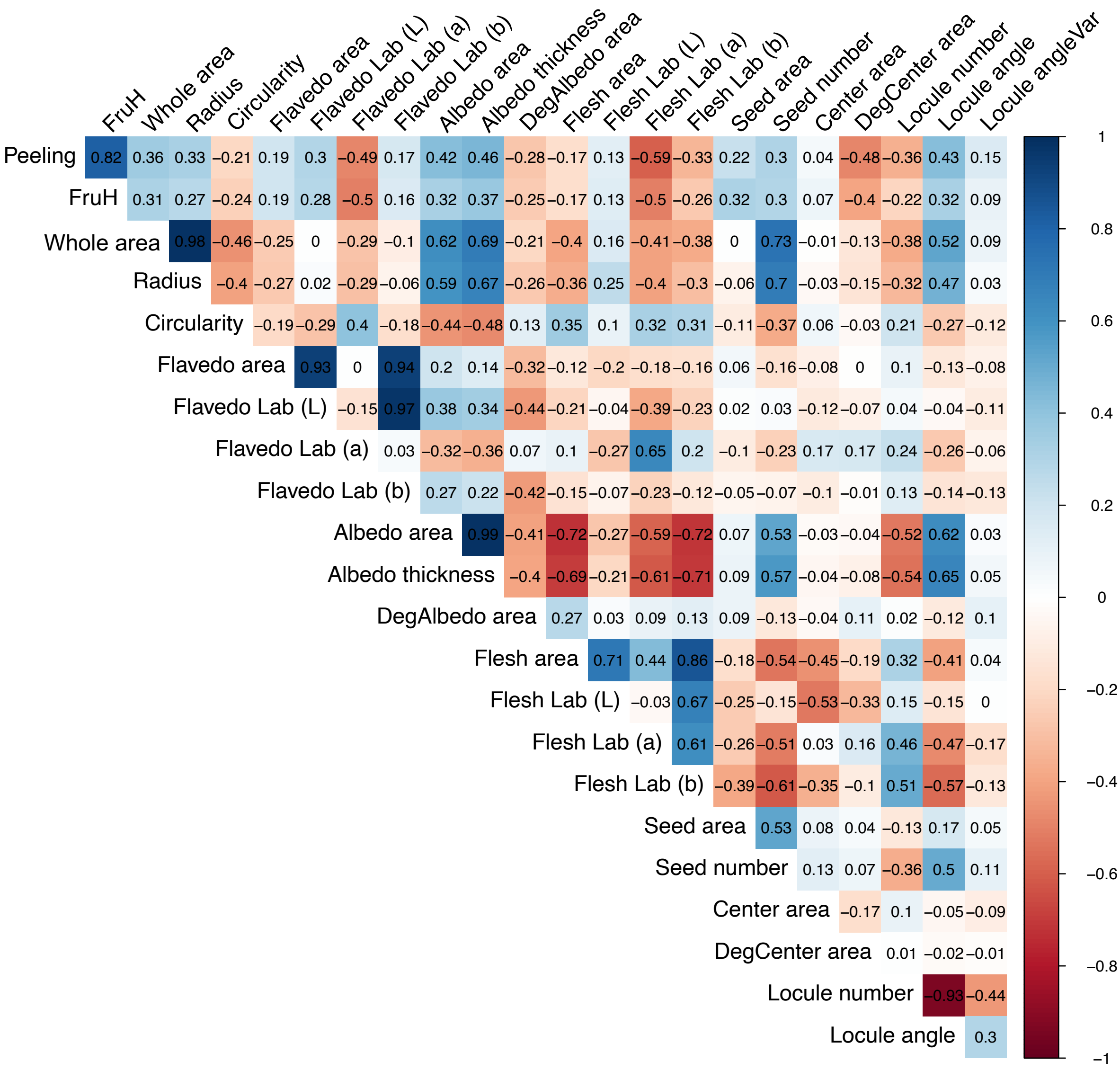

Supplementary Figure 1. Correlation matrix heatmap for fruit morphological features and breeder-evaluated fruit quality traits.

Peeling and FruH are breeder-evaluated fruit quality traits. The remains are fruit morphological features derived from eight different regions of citrus fruit.

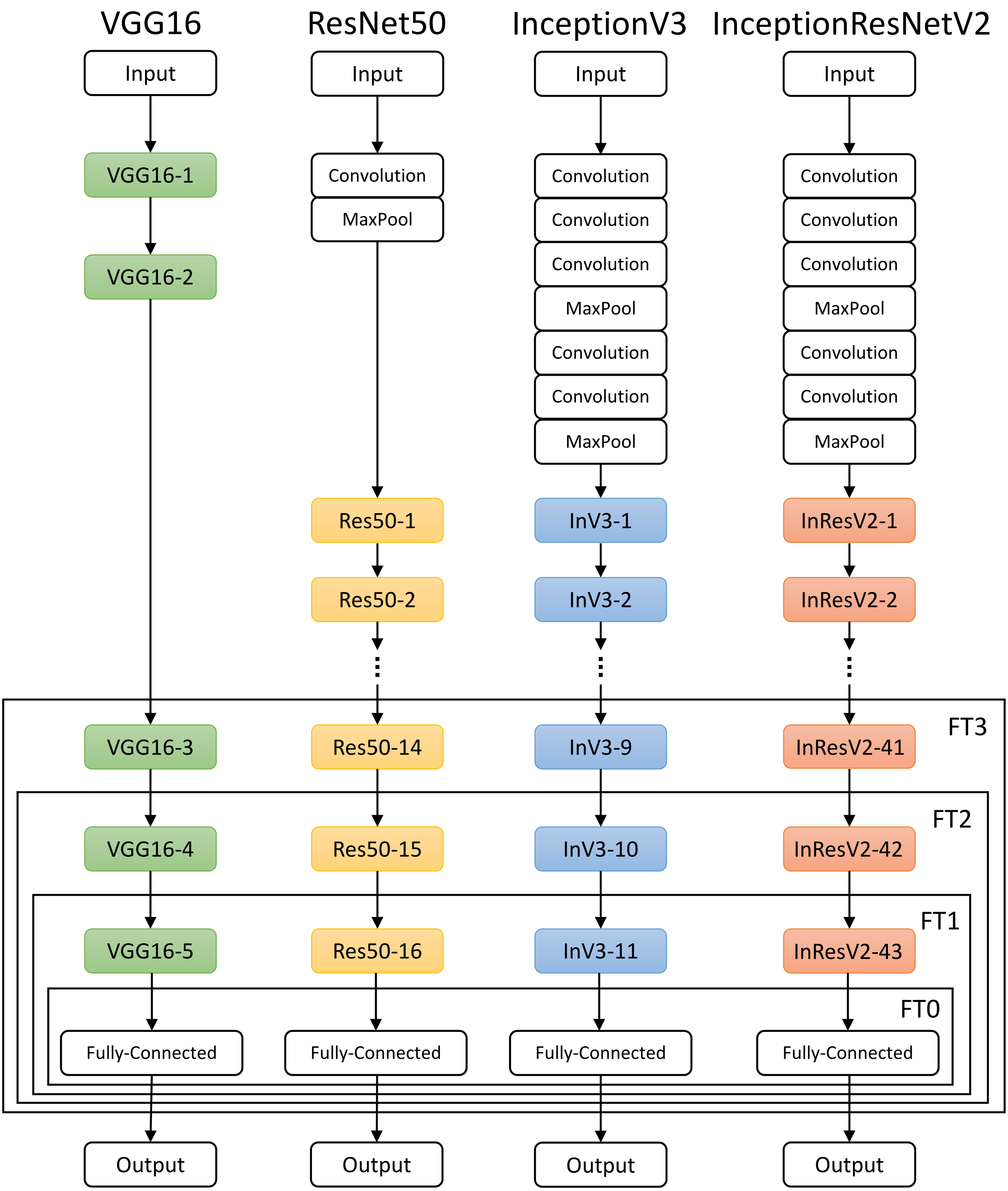

Supplementary Figure 2. Fine-tuning strategy across four deep learning models.
For VGG16 (green modules), one module contains convolutional operations followed by an activation and a pooling layer. For ResNet50 (yellow modules), one module is a residual block, while for InceptionV3 (blue modules), one module is called an inception module. The hybrid of an inception module and a residual connection is regarded as one module for InceptionResNetV2 (orange). FT stands for fine-tuning.

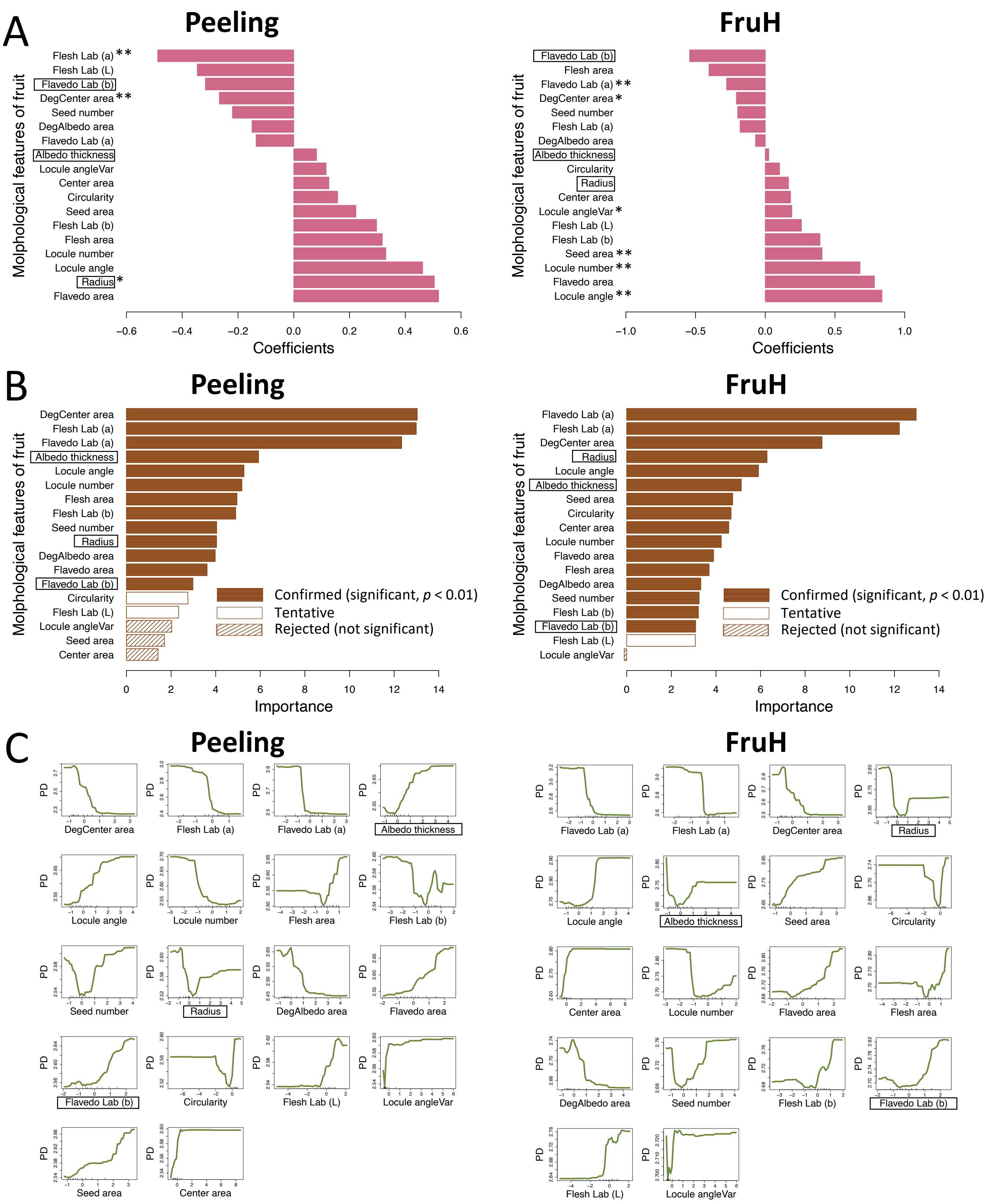

Supplementary Figure 3. Associations between fruit morphological features (including Radius, Flavedo Lab (b), and Albedo thickness instead of Whole area, Flavedo Lab (L), and Albedo area) and breeder-evaluated fruit quality traits using multiple linear regression and random forest. Radius, Flavedo Lab (b), and Albedo thickness were not randomly selected in model constructions to prevent multicollinearity (Figure 3), because they were highly correlated with Whole area (r = 0.98), Flavedo Lab (L) (r = 0.97), and Albedo area (r = 0.99), respectively (Supplementary Figure 1).
(A) Regression coefficients estimated using multiple linear regression (MLR).
Asterisks indicate statistically significant correlations: *$p < 0.05$; **$p < 0.01$. (B) Variable importance in the random forest (RF) model. (C) Partial dependence calculated for the RF model.

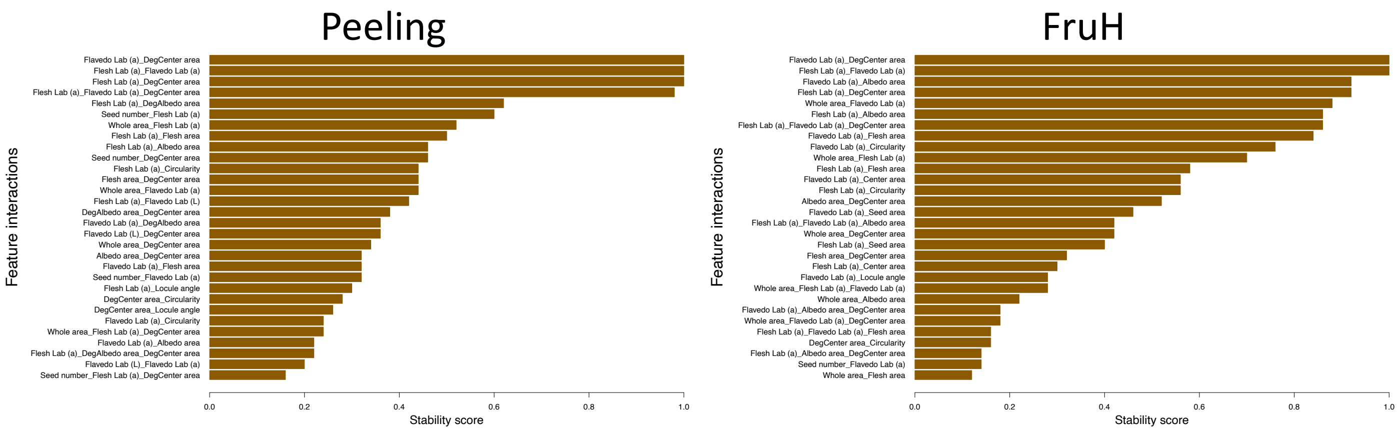

Supplementary Figure 4. Interactions between the explanatory variables (i.e., fruit morphological features) with random forest regression.

Peeling and FruH, which are breeder-evaluated fruit quality traits, are response variables in each regression. Thirty of the most stable interactions between fruit morphological features are shown.

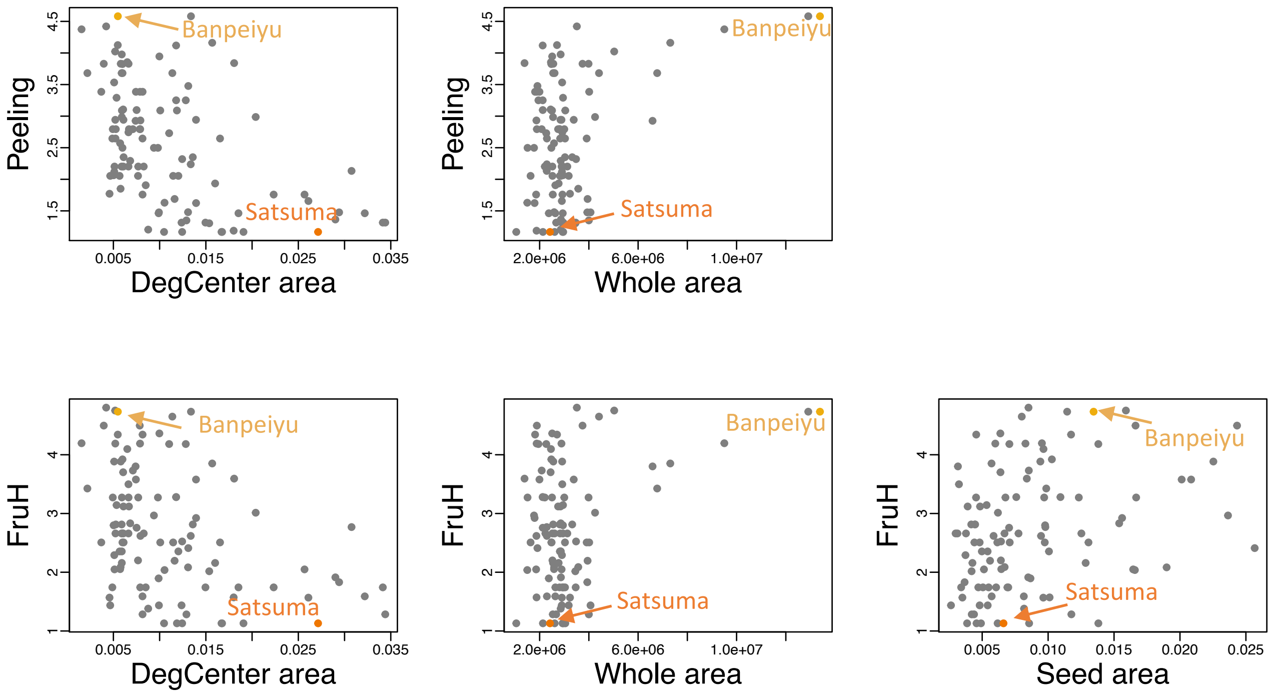

Supplementary Figure 5. Scatter plots of the fruit morphological features and Peeling or FruH. Both DegCenter and Whole areas were significantly correlated with Peeling and FruH in the partial correlation (Figure 4B). In contrast, Seed area was significantly correlated with only FruH (Figure 4B).

Peeling 2013

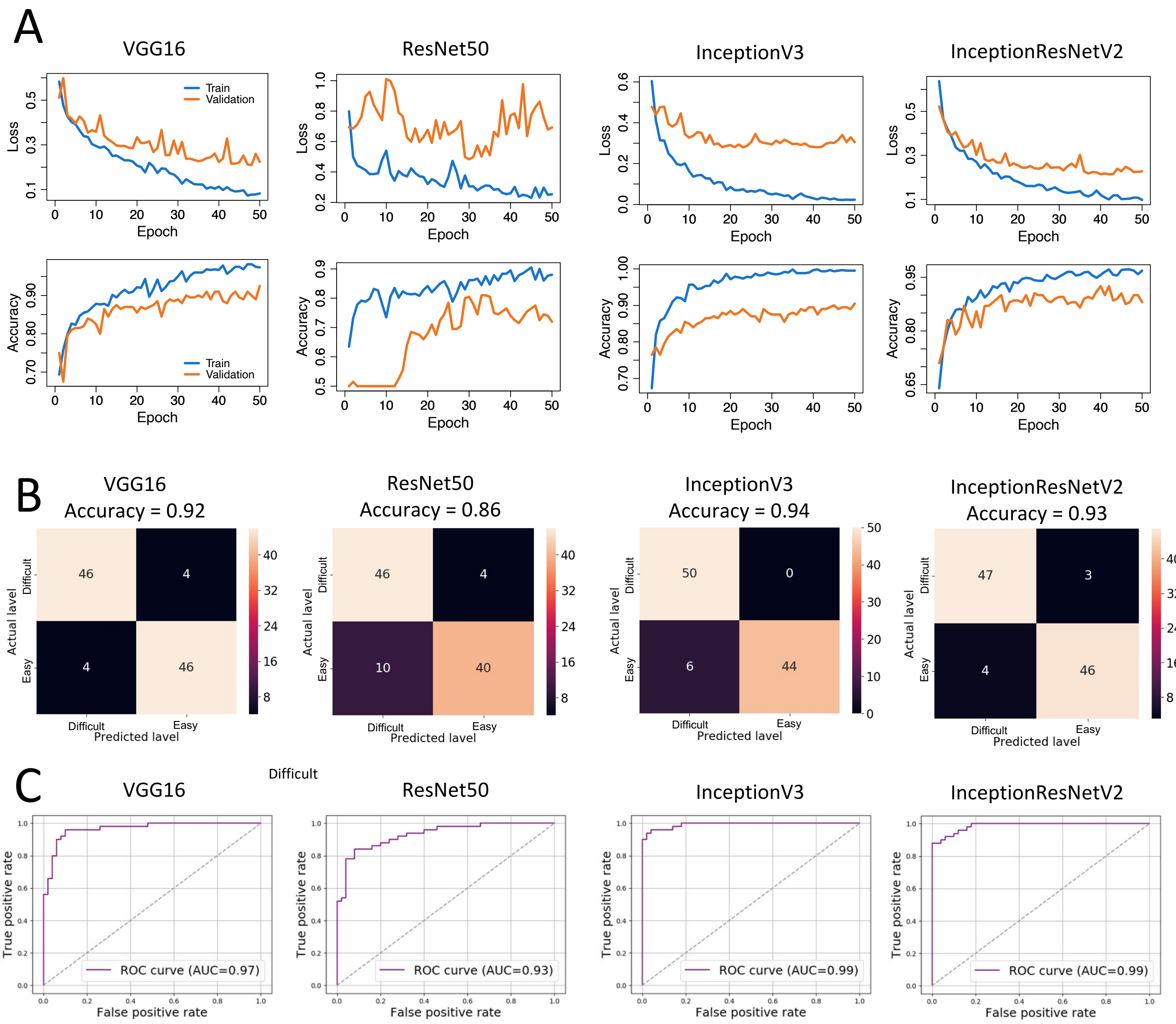

Supplementary Figure 6. Evaluation of the four deep-learning models for Peeling classification using the images of citrus fruits harvested in 2013.
The fine-tuning strategies, which showed the greatest accuracy in classification (Table 3) for each model are shown. If two or more fine-tuning strategies demonstrated the same accuracy, the strategy with the least amount of fine-tuning was selected. (A) Model accuracy and loss curves for 50 epochs.
(B) Confusion matrix. Classification accuracy is shown at the top of the matrix. (C) Receiver operating characteristic (ROC) curve. The value of area under the ROC curve (AUC) is shown in the bottom right.

FruH 2013

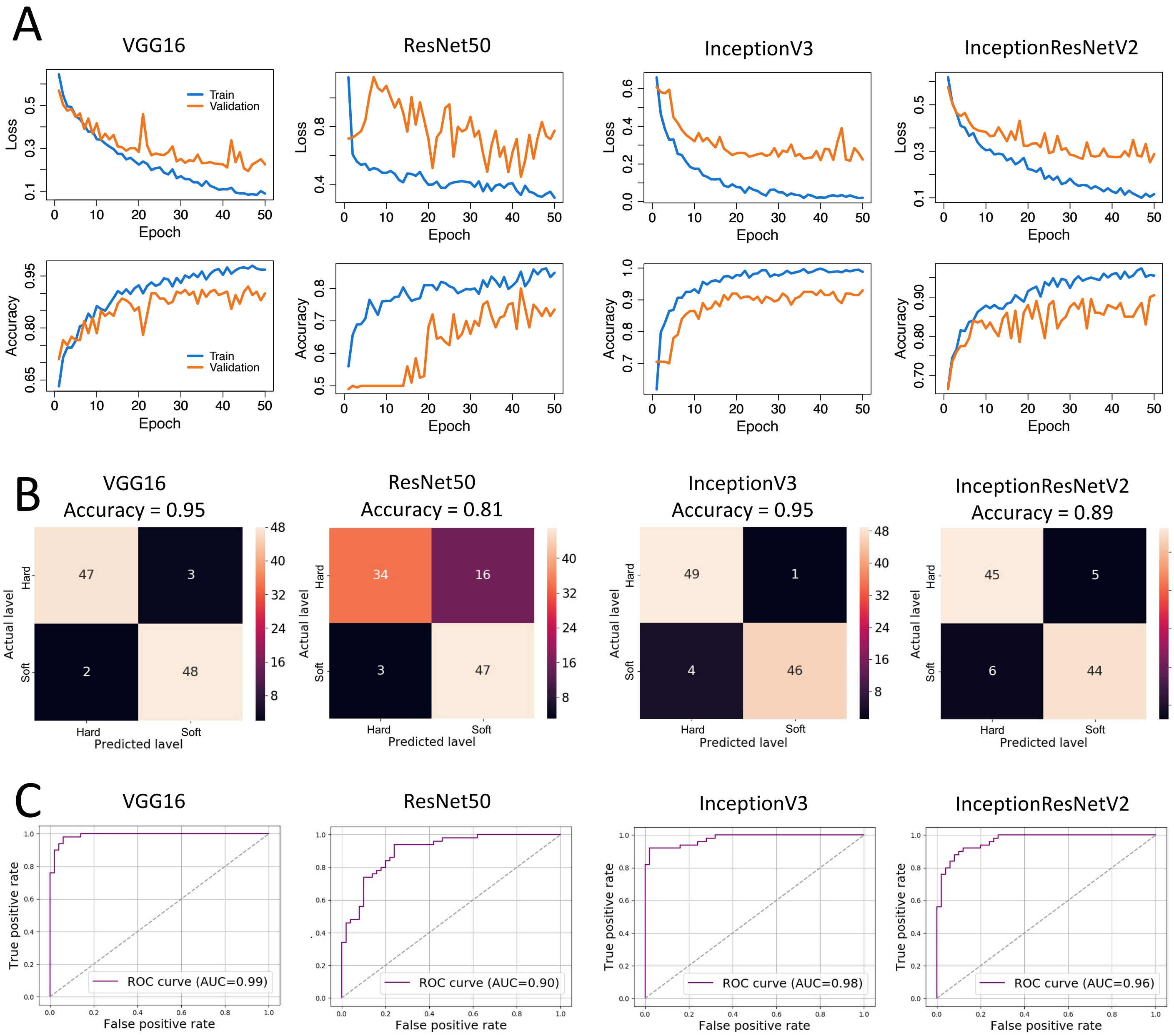

Supplementary Figure 7. Evaluation of the four deep-learning models for FruH classification using the images of citrus fruits harvested in 2013.
The fine-tuning strategies, which showed the greatest accuracy in classification (Table 3) for each model are shown. If two or more fine-tuning strategies demonstrated the same accuracy, the strategy with the least amount of fine-tuning was selected. (A) Model accuracy and loss curves for 50 epochs.
(B) Confusion matrix. Classification accuracy is shown at the top of the matrix. (C) Receiver operating characteristic (ROC) curve. The value of area under the ROC curve (AUC) is shown in the bottom right.

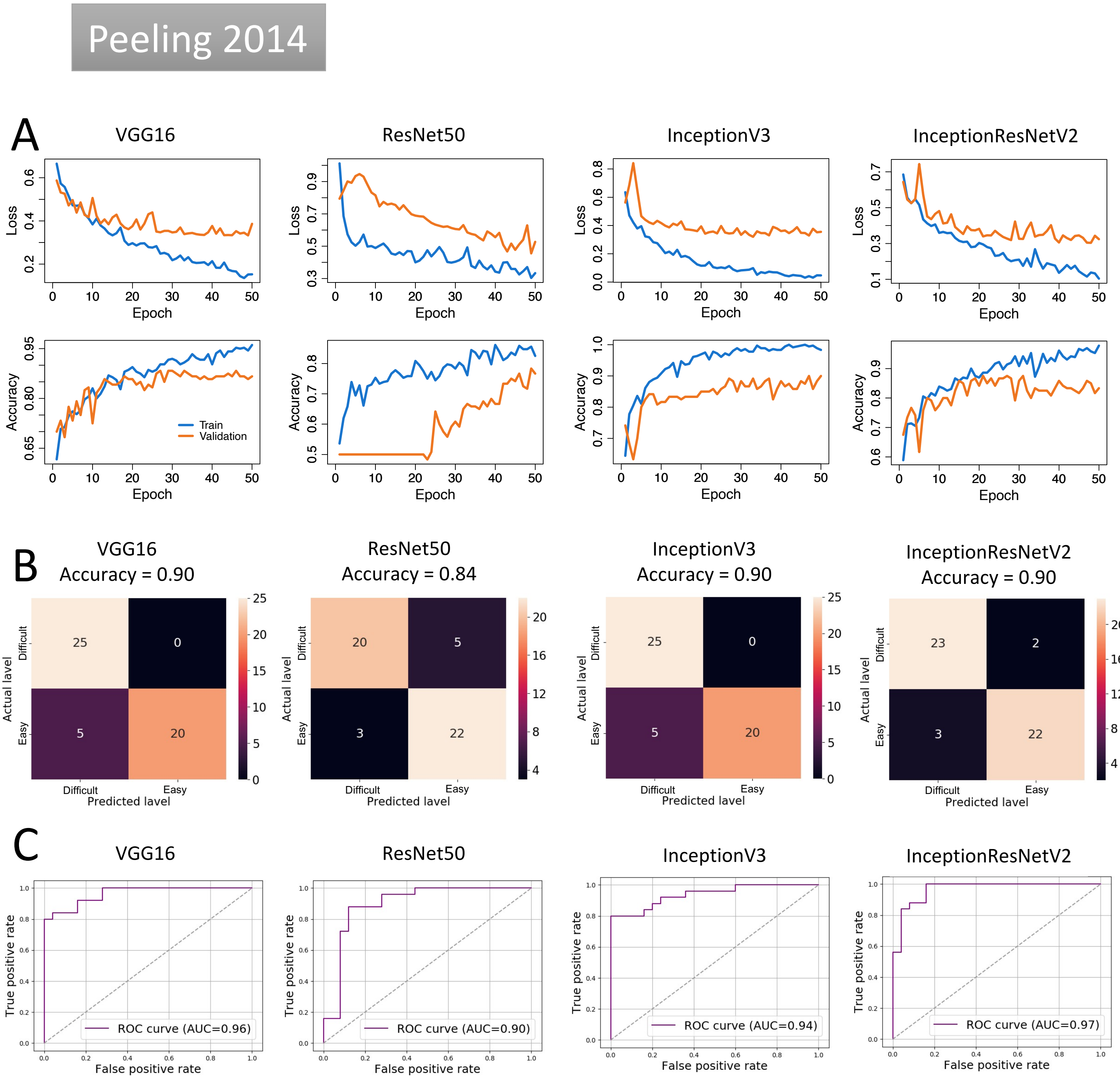

Supplementary Figure 8. Evaluation of the four deep-learning models for Peeling classification using the images of citrus fruits harvested in 2014.

The fine-tuning strategies, which showed the greatest accuracy in classification (Table 3) for each model are shown. If two or more fine-tuning strategies demonstrated the same accuracy, the strategy with the least amount of fine-tuning was selected. (A) Model accuracy and loss curves for 50 epochs.
(B) Confusion matrix. Classification accuracy is shown at the top of the matrix. (C) Receiver operating characteristic (ROC) curve. The value of area under the ROC curve (AUC) is shown in the bottom right.

FruH 2014

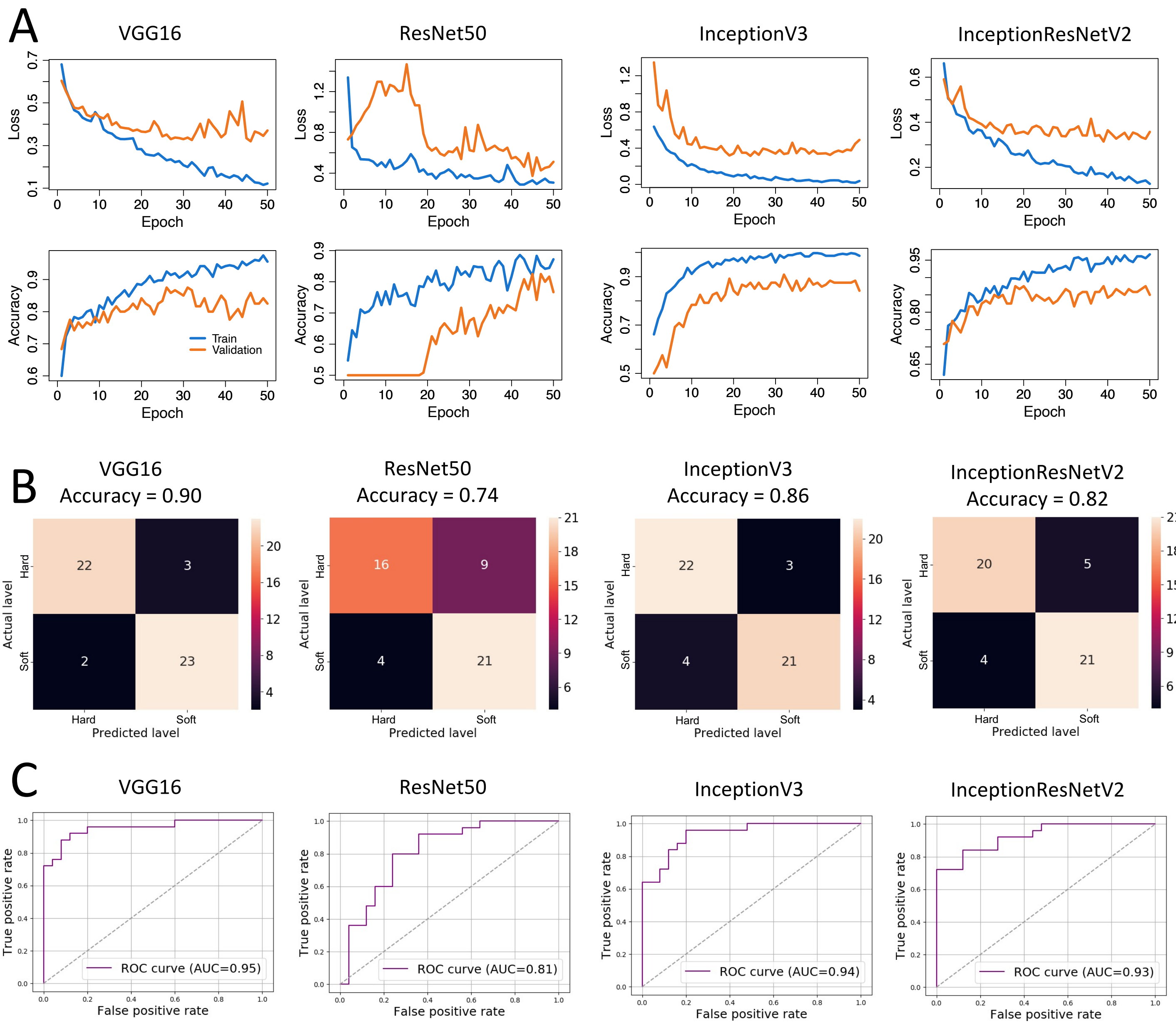

Supplementary Figure 9. Evaluation of the four deep-learning models for FruH classification using the images of citrus fruits harvested in 2014.
The fine-tuning strategies, which showed the greatest accuracy in classification (Table 3) for each model are shown. If two or more fine-tuning strategies demonstrated the same accuracy, the strategy with the least amount of fine-tuning was selected. (A) Model accuracy and loss curves for 50 epochs.
(B) Confusion matrix. Classification accuracy is shown at the top of the matrix. (C) Receiver operating characteristic (ROC) curve. The value of area under the ROC curve (AUC) is shown in the bottom right.

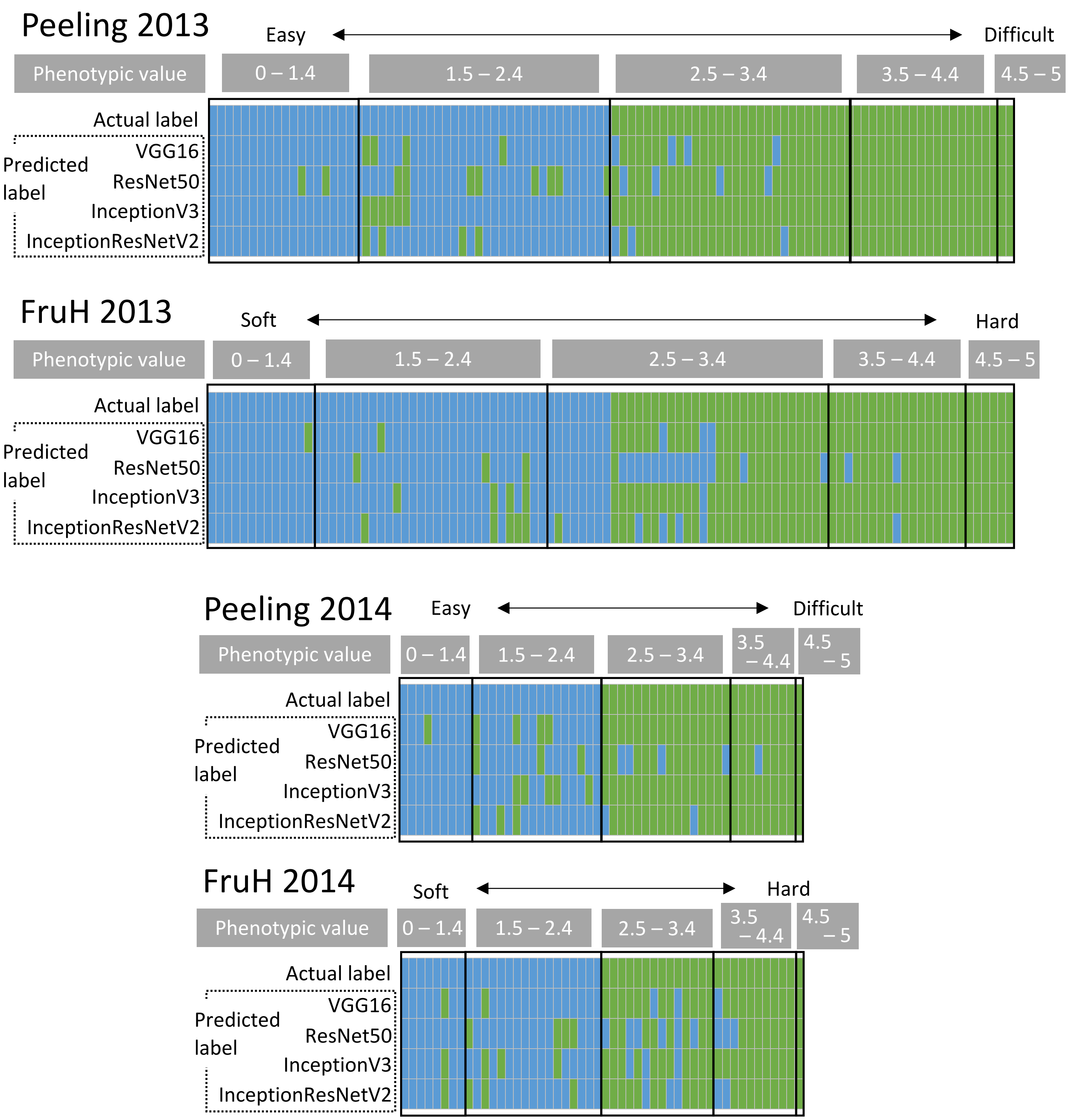

Supplementary Figure 10. Actual and predicted labels for each fruit image in prediction datasets used to evaluate the learned deep learning models.
The binary colors indicate the binary labels, respectively. The fine-tuning strategies, which showed the greatest accuracy in classification (Table 3) for each model are shown. If two or more fine-tuning strategies demonstrated the same accuracy, the strategy with the least amount of fine-tuning was selected.
